# Comprehensive Analysis of β-1,3-Glucanase Genes in Wolfberry and Their Implications in Pollen Development

**DOI:** 10.3390/plants14010052

**Published:** 2024-12-27

**Authors:** Xin Zhang, Pinjie Zheng, Xurui Wen, Zhanlin Bei

**Affiliations:** 1 College of Biological Science and Engineering, North Minzu University, Yinchuan 750021, China; x_zhang@nmu.edu.cn (X.Z.); 20223493@stu.nmu.edu.cn (P.Z.); 18235769035@163.com (X.W.); 2 Key Laboratory of Biodiversity and Ecological Engineering, Ministry of Education, Fudan University, Shanghai 200437, China

**Keywords:** gene expression, glycoside hydrolase family, molecular characterization, evolutionary relationships, transcriptomic profiling

## Abstract

β-1,3-Glucanases (Glu) are key enzymes involved in plant defense and physiological processes through the hydrolysis of β-1,3-glucans. This study provides a comprehensive analysis of the β-1,3-glucanase gene family in wolfberry (*Lycium barbarum*), including their chromosomal distribution, evolutionary relationships, and expression profiles. A total of 58 *Glu* genes were identified, distributed across all 12 chromosomes. Evolutionary analysis revealed six distinct branches within wolfberry and nine distinct branches when compared with *Arabidopsis thaliana*. Expression analysis showed that 45 *Glu* genes were expressed in berries, with specific genes also being expressed in flowers and leaves. Notably, *LbaGlu28* exhibited significant expression during the tetrad stage of pollen development and was localized in the cell wall. These findings provide valuable insights into the functional significance of *Glu* genes in wolfberry, highlighting their roles in development and their potential involvement in reproductive processes, particularly in pollen development.

## 1. Introduction

Glu are a class of enzymes that catalyze the hydrolysis of β-1,3-glucans, which are major components of fungal cell walls and various plant tissues [1,2]. They belong to the pathogenesis-related proteins-2 (PR-2) family and are widely distributed among bacteria, fungi, viruses, and plants such as Arabidopsis, rice, tobacco, and soybean [3,4]. Structurally, β-1,3-glucanases typically comprise an N-terminal signal peptide, a C-terminal domain, and a glycoside hydrolase family 17 (GH17) functional domain, exhibiting diverse isoforms with variations in size, structure, and cellular localization [5]. In biological systems, β-1,3-glucanases play vital roles, primarily acting as defenders against fungal pathogens by breaking down fungal cell wall β-1,3-glucans and showing potent antifungal activity [6,7]. This activity is crucial for protecting plants from infections and is frequently associated with pathogenesis-related (PR) responses. These enzymes participate in both constitutive and induced defense mechanisms, contributing to the hydrolysis of fungal cell wall β-1,3-glucans as a primary defense barrier and releasing immune elicitors during induced defense, thereby enhancing defense responses [6,7]. Beyond defense, β-1,3-glucanases have been implicated in numerous physiological processes in plants, including cell wall remodeling, stress responses, and developmental processes such as pollen development and anther ripening [8,9,10]. Some members are expressed in healthy plant roots and floral tissues, indicating roles in hormonal and developmental regulation beyond defense mechanisms. These enzymes likely influence processes such as cell wall remodeling and morphogenesis [11,12]. In summary, β-1,3-glucanases are pivotal components of both plant defense mechanisms and normal physiological processes, underscoring their importance in plant biology and potential applications in enhancing plant resistance to fungal pathogens.

Wolfberry *Lycium barbarum* L. is a plant species renowned for its medicinal and nutritional properties [13]. Wolfberry has been traditionally used in Chinese medicine for centuries due to its numerous health benefits, including antioxidant properties, immune system support, and potential anti-aging effects [14,15]. In addition to its nutritional value, wolfberry holds significance in agricultural practices and serves as a subject of scientific research. One crucial aspect of wolfberry biology is the development of its pollen, which is essential for successful reproduction and anther formation. Pollen development in wolfberry is intricately regulated, with various genetic factors influencing the process [16,17]. Of particular importance is the degradation of sporopollenin, a major constituent of pollen exine, which is crucial for proper pollen wall formation and subsequent germination. Dysregulation of sporopollenin metabolism can lead to male sterility, significantly impacting reproductive success in wolfberry [16,17,18]. Recent studies have identified several genes involved in pollen development in wolfberry, with β-1,3-glucanases emerging as key players in this process [2,19,20]. β-1,3-glucanases are enzymes known for their role in the degradation of callose, a β-1,3-glucan polymer present in the cell walls of plant tissues. The degradation of callose is essential for pollen development and subsequent release. However, the involvement of β-1,3-glucanases in wolfberry pollen development remains unknown.

In this study, we employed a multifaceted approach to elucidate the landscape of *Glu* genes in wolfberry (*Lycium barbarum*). By utilizing bioinformatics tools and molecular techniques, we identified a total of 58 putative *Glu* genes within the wolfberry genome. Our investigation encompassed comprehensive analyses of their chromosomal distribution, gene structure, conserved motifs, and cis-regulatory elements, providing insights into the evolutionary dynamics and regulatory mechanisms governing *Glu* gene expression in wolfberry. Furthermore, we investigated the involvement of *Glu* in wolfberry reproductive development, shedding light on their functional significance and potential applications in agricultural practices. By employing a combination of molecular biology techniques, bioinformatics analyses, and physiological assays, we seek to deepen our understanding of the molecular mechanisms underlying pollen development in wolfberry as well as its implications for crop yield and quality.

## 2. Results

### 2.1. The Identification of Glu Family Members in Wolfberry and Analysis of the Physicochemical Properties

In this study, we employed a comprehensive approach to identify *Glu* genes within the genome of wolfberry, focusing on the GH17 (PF00332) domain using Hidden Markov Model (HMM) searches with both HMM profiles and BLASTP. Initially, a set of known Glu protein sequences, validated through prior reviews, were used for querying. The candidate Glu protein sequences were further subjected to domain analysis using SMART and batchCD to confirm the presence of the GH17 domain. A total of 58 putative *Glu* genes were identified (Table S1), each designated as *LbaGlu01* to *LbaGlu58* based on their respective chromosomal locations within wolfberry. The table includes detailed information on the chromosomal positions of *Glu* genes, their coding sequence (CDS) lengths, protein lengths, predicted molecular weights, and hydrophilicity. Glu proteins amino acid sequence length ranged from 153 (*LbaGlu17*) to 610 (*LbaGlu14*), and their isoelectric point ranged from 4.79 (*LbaGlu28*) to 9.51 (*LbaGlu33*). Most of their GRAVY values were negative (41/58), indicating that most of them were hydrophilic. This comprehensive analysis provides a foundational understanding of the *Glu* gene landscape in *L. barbarum*, shedding light on their genomic organization and potential functional roles.

### 2.2. Chromosome Localization and Collinearity Analysis of Glu Family Members in Wolfberry

**Figure 1:**
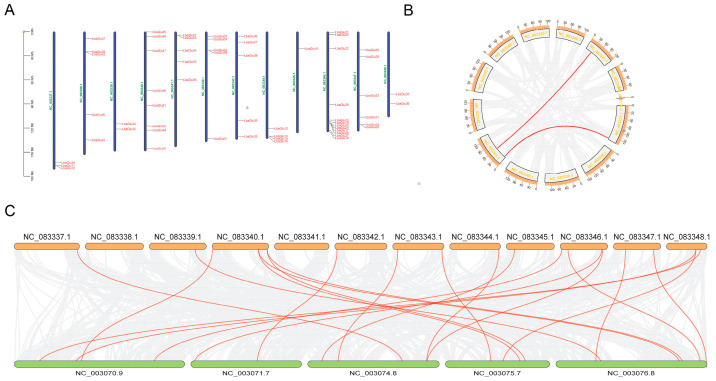
Chromosomal distribution and collinearity analysis of *Glu* family members in wolfberry. (**A**) Chromosomal localization of *Glu* genes on the 12 chromosomes of wolfberry. Each colored bar represents a chromosome, and *Glu* genes are denoted by vertical lines along the chromosomes. (**B**) Collinearity analysis showing replication relationships between *Glu* gene family members within the wolfberry genome. Genes connected by lines represent collinear relationships. (**C**) Inter-species collinearity analysis between wolfberry and *Arabidopsis thaliana*.

The chromosomal distribution of *Glu* family members is depicted in Figure 1A, illustrating their presence across all 12 chromosomes. NC_083346.1 contained the highest number of *Glu* members, with 12 located on this chromosome, while NC_083345.1 had the lowest distribution, with only one *Glu* member. Notably, a majority of *Glu* family members were found in close proximity to telomeric regions. In order to comprehend the evolutionary internal relevancies of *Glu* family genes, the collinearity relationships between *Glu* genes were identified (Figure 1B). Within the species, *Glu* gene family members exhibited only two collinear relationships, specifically between *LbaGlu05* and *LbaGlu06* and *LbaGlu08* and *LbaGlu10*. This analysis offers insights into the genomic organization of *Glu* genes in wolfberry, highlighting both their chromosomal distribution and replication relationships. Furthermore, we employed MCScanX to conduct inter-species collinearity analysis between wolfberry and *Arabidopsis thaliana* (Figure 1C). Our analysis revealed significant orthologous relationships between *Glu* genes of wolfberry and those of Arabidopsis, identifying 21 orthologous pairs. These gene loci above in both wolfberry and Arabidopsis were located unevenly at different chromosomes. These findings underscore the conserved nature of *Glu* genes across evolutionary distances, suggesting a shared structural and functional similarity between *Glu* genes in wolfberry and Arabidopsis.

### 2.3. Gene Structure and Conserved Motif Analysis of Glu Genes

**Figure 2:**
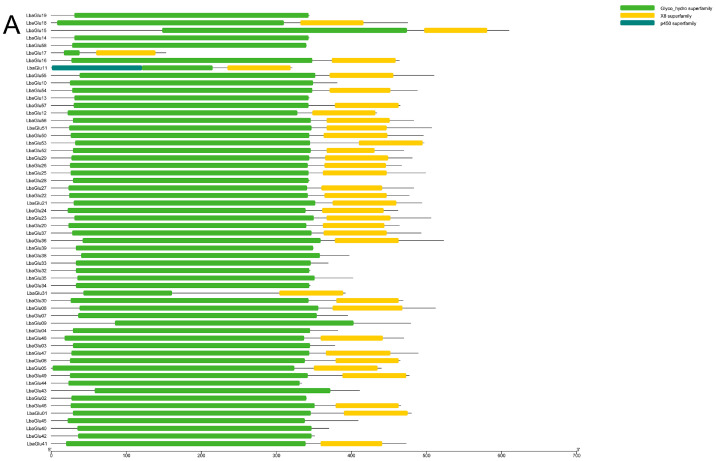

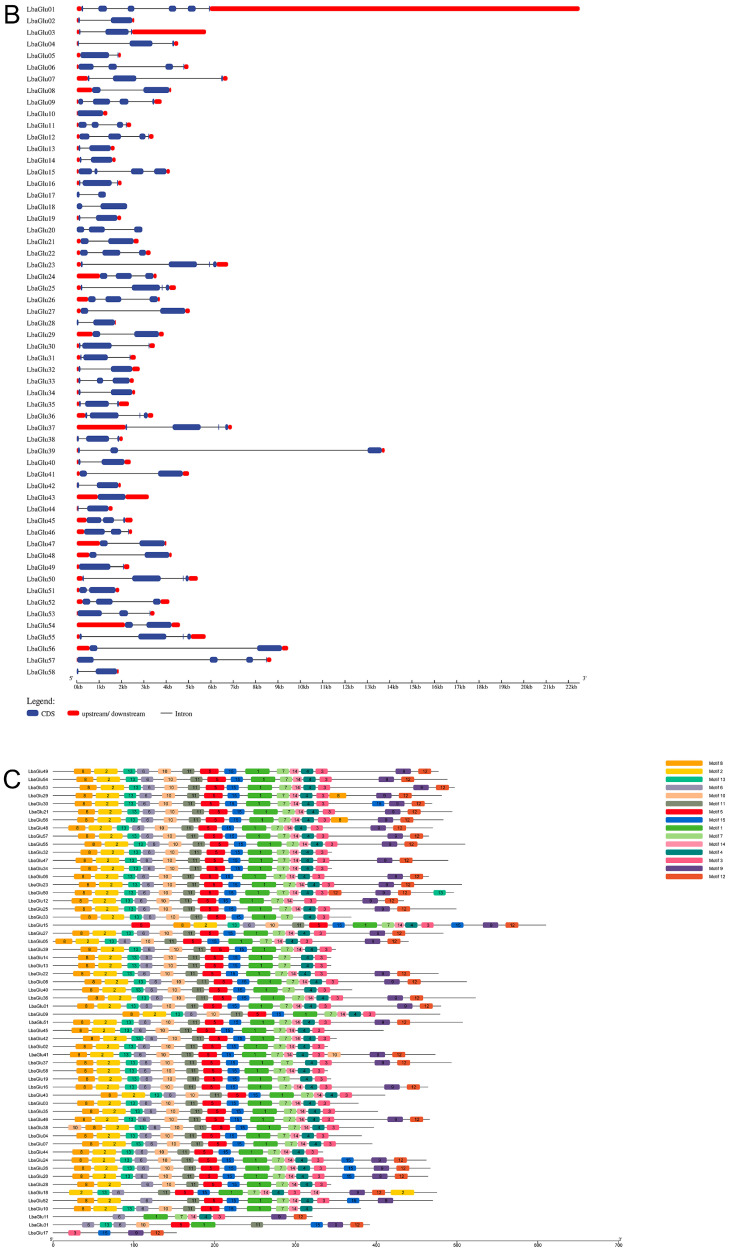
Gene structure and conserved motif analysis of *Glu* family members in wolfberry. (**A**) Conserved motif analysis revealing the presence of up to 15 motifs among *Glu* genes, with most genes possessing motifs 1 and 7. (**B**) Gene structure analysis showing the exon–intron organization of *Glu* genes. The number of exons and introns varies among different *Glu* family members. (**C**) Gene structure and conserved motif analysis of *Glu* family members in wolfberry.

To better understand the evolution and structural diversity of the *Glu* gene family, the evolutionary relationships with conserved motifs, promoter elements, and gene structure were evaluated in wolfberry. In terms of conserved motifs, we visualized the results from batchCDD analysis, which revealed the presence of the GH17 domain in all 58 *Glu* genes (Figure 2A). Further structural domain analysis was conducted using DNAMAN, where *Glu* gene sequences were categorized into two groups based on the presence or absence of the X8 domain. The results of this analysis are presented in the Supplementary Figures S1 and S2. Next, we performed gene structure analysis utilizing the Gene Structure Display Server (GSDS) for the identified 58 family genes. As shown in Figure 2B, 25 members of the *Glu* gene family had 2 exomes and 1 intron, accounting for 43.1% of the overall *Glu* gene family. Eighteen *Glu* gene family members had 3 exomes and 2 introns, accounting for 31% of the overall *Glu* gene family. Thirteen *Glu* gene family members had 4 exomes and 3 introns, accounting for 22.4% of the overall *Glu* gene family. *LbaGlu10* and *LbaGlu43* had only one exome, and *LbaGlu01* had 6 exomes. In general, most of the *Glu* family members had 2–4 exomes and 1–3 introns. Subsequently, we analyzed the conserved motifs of *Glu* gene sequences using the MEME suite (Figure 2C). A maximum of 15 motifs were identified, with an optimal motif width set between 3 and 50 base pairs. Notably, all genes except *LbaGlu17* had a motif1 domain. Moreover, with the exception of *LbaGlu17* and *LbaGlu31*, all genes possessed both motif1 and motif7 domains. These findings provide insights into the conservation patterns within the *Glu* gene family.

### 2.4. Cis-Regulatory Element Analysis of Glu Genes in Wolfberry

**Figure 3:**
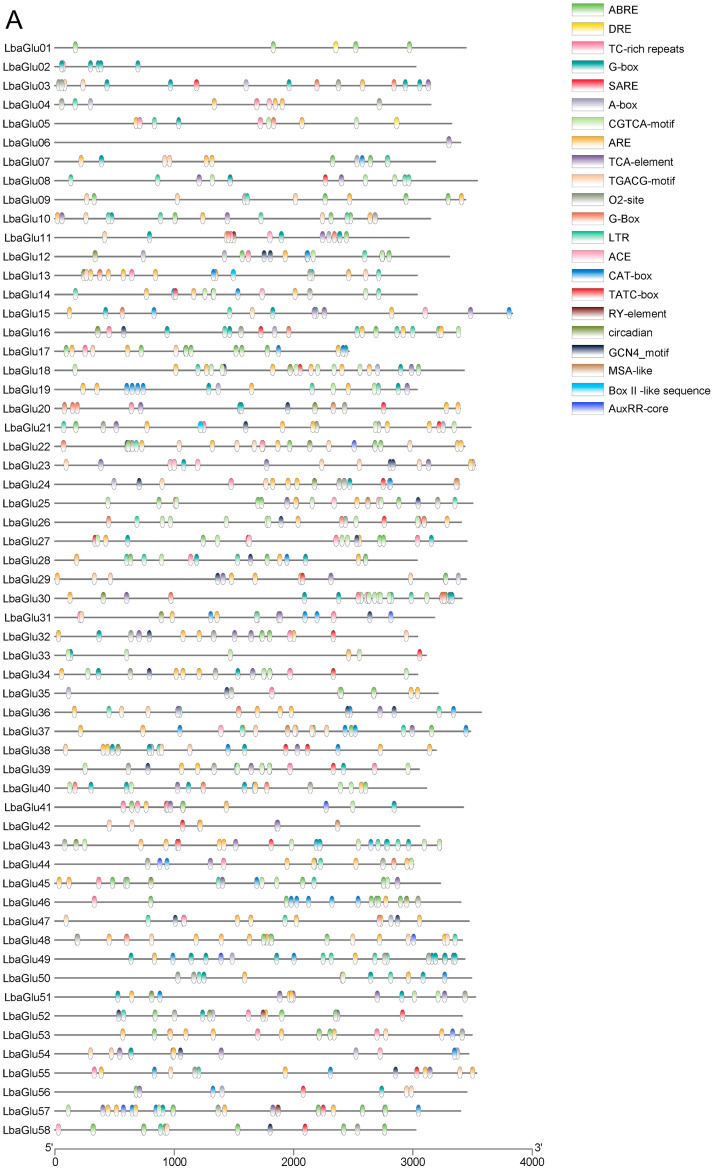

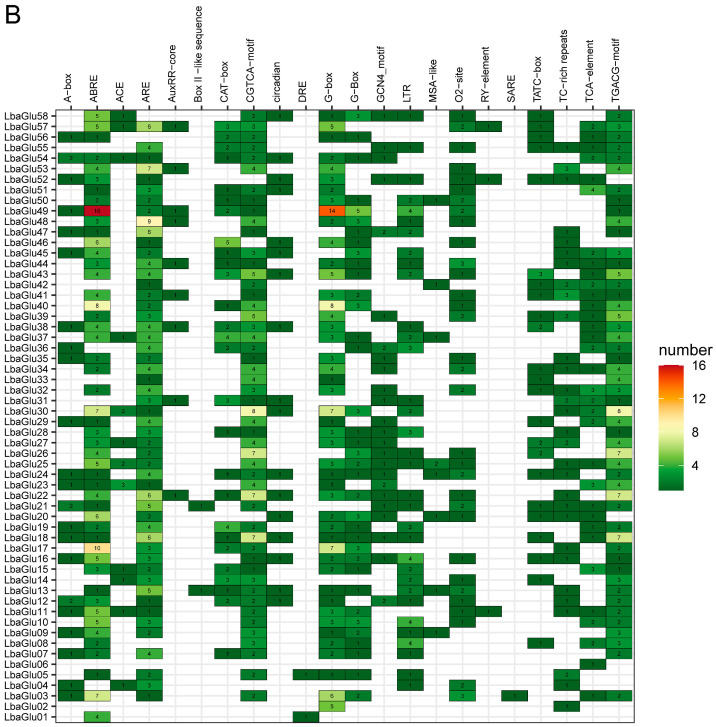
Prediction of cis-acting elements of *Glu* family members in wolfberry. (**A**) Heatmap visualization of the distribution patterns of cis-regulatory elements among *Glu* genes. Elements are classified into 22 distinct categories based on their sequence features and putative functions. (**B**) Comparison of cis-regulatory element frequencies, highlighting higher occurrences of ARBE and G-box elements in specific *Glu* gene family members.

We analyzed the 2000 bp upstream regions of 58 *Glu* genes using the Plant CARE program to identify potential cis-regulatory elements related to transcriptional activity and responses to biotic and abiotic stresses in wolfberry. The analysis revealed 69 distinct motifs, which were categorized into promoter-related, light-responsive, hormone-related, abiotic stress-responsive, and biotic stress-responsive elements (Figure 3A). Promoter-related cis-elements covered the largest area, accounting for 67.9% of the identified motifs. TATA-box and CAAT-box were abundantly present across all 58 *Glu* genes, functioning as binding sites for transcription factors to initiate transcription.

For subsequent heatmap visualization, we excluded the highly prevalent TATA-box and CAAT-box motifs, as well as motifs unrelated to our study. This resulted in the retention of 1228 motifs for visualization (Figure 3B). Among these retained motifs, 21 types of cis-elements were identified. The cis-acting regulatory element involved in MeJA-responsiveness covered the largest area at 23.6%, followed by light-responsive cis-acting regulatory elements at 17%, abscisic acid-responsive cis-elements at 14.6%, anaerobic induction-essential cis-elements at 13.1%, and low-temperature-responsive cis-elements at 4.8%. Specifically, the primary cis-elements involved in MeJA-responsiveness were the CGTCA-motif and TGACG-motif; the major light-responsive cis-element was the G-box; the key abscisic acid-responsive cis-element was ABRE; the essential cis-element for anaerobic induction was ARE; and the main low-temperature-responsive cis-element was LTR. These findings provide a crucial basis for further investigation into the transcriptional regulatory mechanisms of *Glu* genes in goji under various stress conditions.

### 2.5. Evolutionary Relationships Analysis of Glu Gene Family

**Figure 4:**
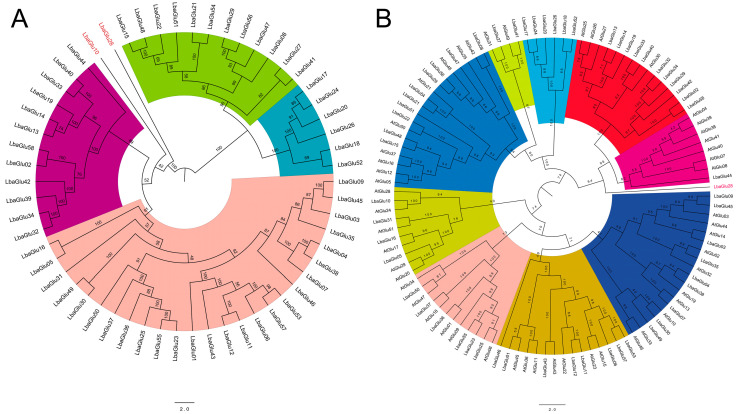
Evolutionary relationship analysis of *Glu* genes in wolfberry and interspecific comparison with *Arabidopsis thaliana*. (**A**) Evolutionary tree of *Glu* genes within wolfberry, categorized into 6 branches based on DNA sequence similarity. (**B**) Interspecific evolutionary analysis comparing *Glu* genes from wolfberry (*Lycium barbarum*) and *Arabidopsis thaliana*. The tree reveals 9 distinct branches, indicating evolutionary divergence and potential common ancestry between the *Glu* gene families of the two species.

In order to analyze the evolutionary relationship of the *Glu* gene family in wolfberry, an evolutionary tree was constructed using the DNA sequences of *Glu* genes. As shown in Figure 4A, the evolutionary tree of *Glu* genes within wolfberry could be divided into 6 branches, suggesting intricate evolutionary diversification within the species. Furthermore, we also performed an interspecific evolutionary analysis of *Glu* genes in wolfberry and *Arabidopsis thaliana*. Identification of the *Glu* gene family in *Arabidopsis thaliana* was conducted using the previously mentioned methodology, resulting in the identification of 51 *Glu* family genes designated as *AtGlu01* to *AtGlu51* based on their chromosomal locations. An evolutionary tree was constructed using the 58 genes from wolfberry and the 51 genes from *Arabidopsis thaliana* (Figure 4B). The analysis revealed 9 distinct branches, indicating the evolutionary divergence and potential common ancestry between *Glu* gene families of the two species. It is worth noting that *LbaGlu28* occupies basal branches on both trees, suggesting its antiquity and value for research.

### 2.6. Expression of Glu Gene Family Members in Different Tissues of Wolfberry

**Figure 5:**
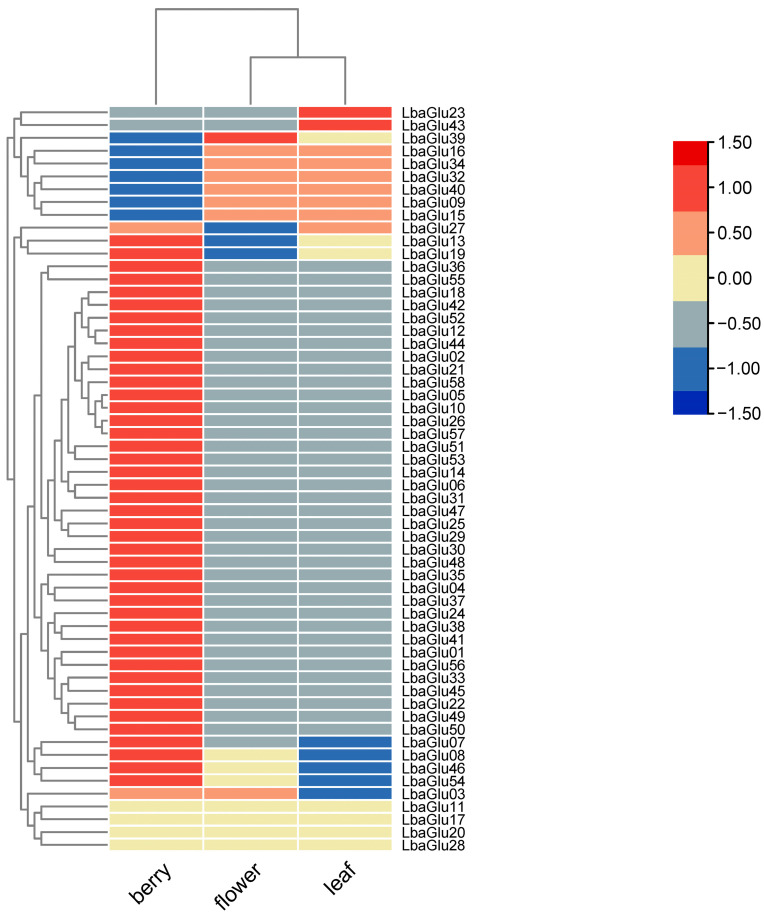
Expression of *Glu* gene family members in different tissues of wolfberry. Heatmap representation of *Glu* gene expression across various tissues, including flowers, leaves, and berries. Tissue-specific expression patterns indicate potential roles of *Glu* genes in different developmental processes within the plant.

The expression patterns of genes in different tissues impacted their function. In order to analyze the putative functions of *Glu* genes in wolfberry development, the expressions of *Glu* gene family members in the berry, flower, and leaf of wolfberry were analyzed using qRT-PCR (Figure 5). The results showed that *Glu* genes exhibited different expression patterns in different tissues. It was evident that the berry exhibited a broad spectrum of *Glu* gene expression, with approximately 45 *Glu* genes being expressed in this tissue. A total of 8 and 9 *Glu* genes were, respectively, expressed in the flower and leaf of wolfberry. Interestingly, the genes *LbaGlu16*, *LbaGlu34*, *LbaGlu32*, *LbaGlu40*, *LbaGlu09*, and *LbaGlu15*, which were expressed in the flower and leaf of wolfberry, showed no expression in the berry. This suggested a tissue-specific regulation of these genes, possibly indicating distinct roles in flower and leaf development rather than berry development. Additionally, the gene *LbaGlu39* demonstrated exclusive expression in flowers, hinting at its potentially pivotal role in flower growth and development. Conversely, genes such as *LbaGlu27*, *LbaGlu23*, and *LbaGlu43* exhibited expression solely in the leaves. These findings provided valuable insights into the tissue-specific expression patterns of *Glu* genes in wolfberry, shedding light on their putative functions in different developmental processes within the plant.

### 2.7. The Potential Role of the Glu Gene in Wolfberry Reproductive Development

Our laboratory’s research primarily focuses on investigating the molecular mechanisms of male sterility in *L. barbarum*. We aim to investigate whether the *Glu* genes played a role in the pollen development of wolfberry. First, we investigated the molecular basis of male sterility in *L. barbarum* using the fertile cultivar Ningqi 1 (NQ1) and the male-sterile cultivar Ningqi 5 (NQ5) from Ningxia, China.

**Figure 6:**
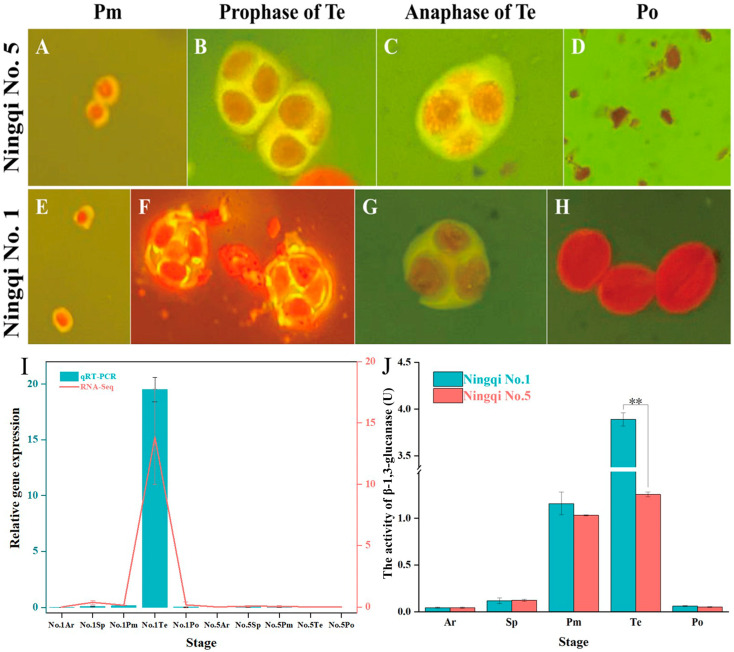
The potential role of *Glu* genes in wolfberry reproductive development. Microscopic observation of anther development stages in fertile (NQ1, **E**–**H**) and male-sterile (NQ5, **A**–**D**) wolfberry cultivars, revealing differences in sporopollenin metabolism and pollen grain formation. (**I**) Transcriptomic analysis and qRT-PCR identifying differential expression of *LbaGlu28* between fertile and male-sterile anthers. (**J**) Enzymatic assays demonstrating glucanase activity patterns in NQ1 and NQ5 anthers, indicative of aberrant sporopollenin metabolism in the male-sterile cultivar. ** indicates a highly significant difference (*p* ≤ 0.01).

As shown in Figure 6E–H, using the squash technique, we extensively observed the developmental stages of microspores within the pollen sacs of wolfberry from Ningxia. These stages were categorized into five periods: sporogenous cell stage (Ar, 1.3 mm), sporocyte stage (Sp, 1.31–1.89 mm), pollen mother cell stage (Pm, 1.90–2.93 mm), tetrad stage (Te, 2.94–4.0 mm), and pollen grain stage (Po, 4.01–5.0 mm). Microscopic analysis revealed distinct differences in exine structure and sporopollenin metabolism during anther development between NQ1 and NQ5 (Figure 6A–H). During the Pm stage, both cultivars exhibited sporopollenin deposition around the pollen mother cells. However, in the early tetrad stage, NQ1 displayed bright yellow tetrads with well-defined sporopollenin layers under fluorescence microscopy, indicating thick sporopollenin deposition. In contrast, sporopollenin deposition in NQ5 appeared markedly different from NQ1. Later in the anther development, NQ1 exhibited a thinning sporopollenin layer during the late tetrad stage, with sporopollenin degradation observed, exposing microspores within the pollen sac. Conversely, NQ5 showed no obvious changes in sporopollenin deposition between the early and late tetrad stages. Subsequent examination during pollen grain release revealed mature red pollen grains with visible germination furrows in NQ1, while NQ5 exhibited fragmented structures under microscopic observation.

Transcriptomic analysis identified a single homologous *Glu* gene, *LbaGlu28*, showing differential expression between NQ1 and NQ5 anthers. The expression of *LbaGlu28* was spatially and temporally specific, with predominant expression during the tetrad stage in NQ1 but remaining low or negligible in NQ5 (Figure 6I). Enzymatic assays further revealed distinct glucanase activity patterns between NQ1 and NQ5 anthers. *LbaGlu28* exhibited elevated enzymatic activity during the sporogenous cell stage in NQ1, maintaining high levels throughout anther development until the pollen grain stage. In contrast, NQ5 displayed consistently lower glucanase activity, with a decreasing trend observed during the tetrad stage (Figure 6J).

These results collectively suggest that abnormal sporopollenin metabolism observed in NQ5 may be attributed to dysregulated expression of the *LbaGlu28* gene.

### 2.8. Subcellular Localization of LbaGlu28 Protein in Plant Cells

**Figure 7:**
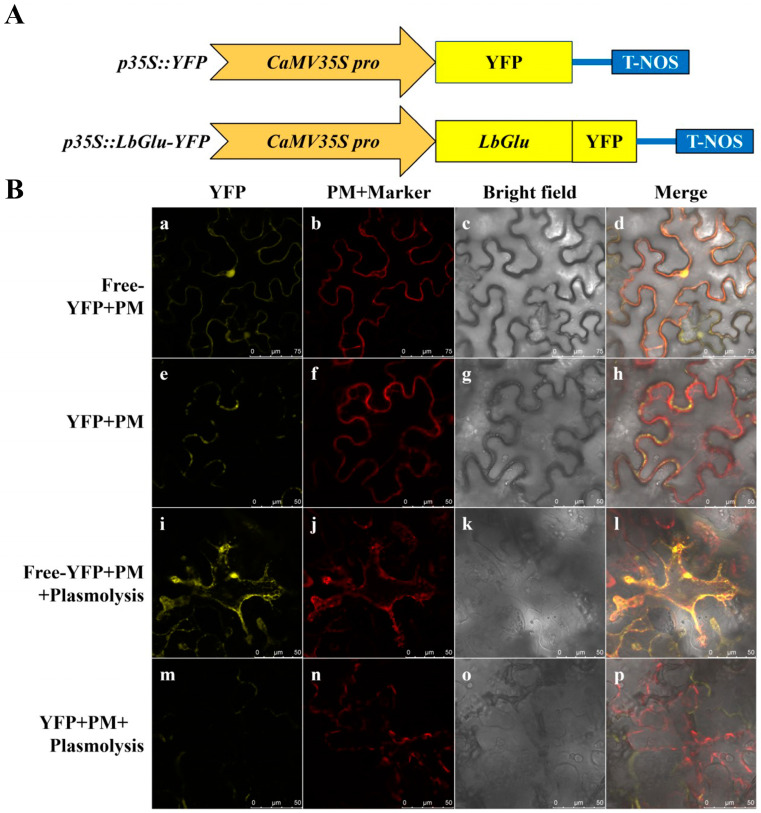
Subcellular localization of LbaGlu28 protein in wolfberry cells. (**A**) Schematic representation of the recombinant vector p35S:LbaGlu28:YFP used for subcellular localization analysis. (**B**) Confocal microscopy images showing the subcellular localization of LbaGlu1:YFP fusion protein in wolfberry cells. Yellow fluorescence indicates the presence of LbaGlu28 protein at the cell wall and plasma membrane, confirmed by the absence of co-localization with the red cell membrane marker during plasmolysis.

Research has shown that Glu proteins in plants are predominantly localized in the cell wall or extracellular space. To investigate the subcellular localization of LbaGlu28, the termination codon of the *LbaGlu28* gene was removed and it was fused with the yellow fluorescent protein (YFP), generating the recombinant vector p35S:LbaGlu28:YFP (Figure 7A). YFP emits a yellow fluorescence signal under confocal laser scanning microscopy. After three days of infiltration, observation under the microscope revealed that YFP was present at both the cell wall and plasma membrane (Figure 7B a–h). To ascertain the subcellular localization of the LbaGlu28 protein, a cell membrane marker was used to label the plasma membrane, which displayed red fluorescence. Under the microscope, during plasmolysis, the red cell membrane did not co-localize with the yellow fluorescence signal from the targeted gene, and LbaGlu28:YFP was observed outside (Figure 7B i–p). This indicated that the LbaGlu28 protein is localized to the cell wall. As outlined in the introduction of this paper, Glu typically participates in the degradation of callose, which is a cell wall polymer primarily composed of glucose linked by β-1,3 bonds. The experimental results confirm the functional role of LbaGlu28 protein in the cell wall.

### 2.9. The Prediction of Three-Dimensional Structures and Signaling Network of LbaGlu28

**Figure 8:**
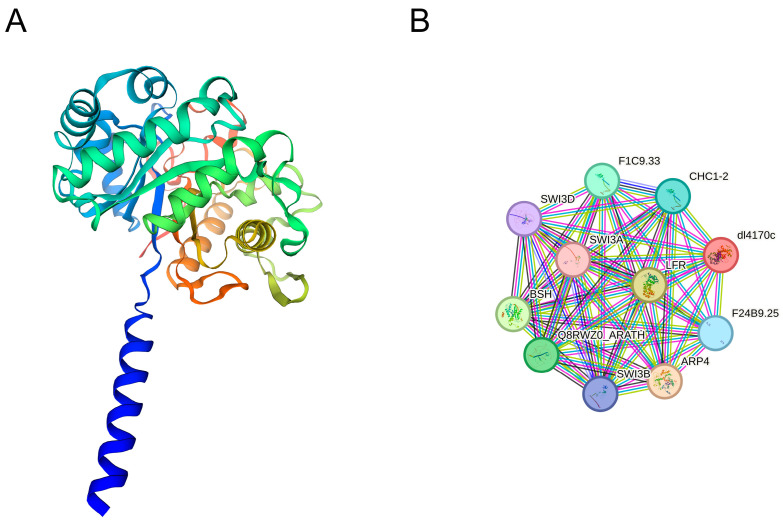
Prediction of three-dimensional structures and signaling network of Glu proteins. (**A**) Predicted three-dimensional structure of LbaGlu28 generated using homology modeling. The structure provides insights into the spatial arrangement of amino acids within the protein. (**B**) Protein–protein interaction (PPI) network analysis predicting potential interactions between proteins and LbaGlu28, highlighting their functional associations within cellular processes.

The structure of a protein, as encoded by its corresponding gene, plays a crucial role in determining its function. Homology modeling represents a valuable approach for predicting the three-dimensional structure of a target protein, leveraging the amino acid sequence to model against experimentally validated protein structures. In our study, we employed SWISS-MODEL to predict the 3D structures of LbaGlu28. Through rigorous evaluation using online software, the accuracy of the 3D structures obtained for LbaGlu28 was confirmed. The template used was A0A1U7XLE8 (A0A1U7XLE8_NICSY) *Nicotiana sylvestris* (Wood tobacco) (South American tobacco). The method used was AlphaFold v2, with the oligomeric state being monomer. It contained the structural domain of Glucan endo-1,3-beta-glucosidase-like isoform X1 and did not contain any ligands, with the GMQE value being 0.94. Figure 8A shows the predicted three-dimensional structure of LbaGlu28.

Furthermore, we conducted protein–protein interaction (PPI) network analysis predictions for LbaGlu28 using the STRING database, with *Arabidopsis thaliana* selected as the model organism. STRING analysis allowed for the prediction of potential interactions among the Glu proteins, shedding light on their functional associations within cellular processes. The predicted signaling network of LbaGlu28 (T4L20.60 in *Arabidopsis thaliana*) is shown in Figure 8B. Function prediction of the STRING database showed that these proteins were mainly involved in the chromatin remodeling, chromatin organization, and regulation of transcription by RNA polymerase II. The results of this network analysis offered valuable information for further exploration of *Glu* gene function and regulation in plants.

## 3. Discussion

In the present study, we performed a comprehensive analysis of the genomic organization, evolutionary relationships, gene structure, expression patterns, and potential functional roles of the *Glu* gene family in wolfberry. The identification of 58 putative *Glu* genes across all 12 chromosomes in wolfberry, designated as *LbaGlu01* to *LbaGlu58*, highlights the richness of this gene family within the genome. Evolutionary analysis revealed intricate evolutionary diversification within the *Glu* gene family in wolfberry, with the evolutionary tree depicting six distinct branches. Moreover, interspecific evolutionary analysis with *Arabidopsis thaliana* identified 21 orthologous pairs of *Glu* genes. *LbaGlu28* occupies basal branches on both trees, suggesting its antiquity and evolutionary importance, warranting further investigation into its functional roles. In 2023, Lui et al. reported 14 full-length sequences of the *Glu* gene in *Hevea brasiliensis* by using the GH17 domain as an identifying feature [21]. Similarly, evolutionary analysis showed the clustering of *Glu* genes into six major clades (I–VI) of *Hevea brasiliensis* based on the domains [21]. Xu et al. identified a total of 67, 68, 130, and 158 *Glus* in four sequenced cotton species: *Gossypium raimondii* (D5), *G. arboreum* (A2), *G. hirsutum acc*. TM-1 (AD1), and *G. barbadense* acc. 3–79 (AD2), respectively [22]. These *GLUs* were classified into eight subfamilies (A–H) due to similar domain architecture and intron/exon structure within each subfamily [22]. Doxey et al. (2007) also identified 50 *Glu* gene sequences from Arabidopsis and categorized them into five classes based on the diversity of the C-terminal region [5].

Our results, combined with collinearity analysis, demonstrated evolutionary divergence and potential common ancestry between the *Glu* gene families of the two species. Gene structure analysis revealed variability in the exon–intron organization among *Glu* family members, with most genes possessing 2–4 exons and 1–3 introns. Additionally, conserved motif analysis identified common motifs shared among *Glu* genes, providing insights into their functional conservation. Cis-acting element analysis uncovered a diverse repertoire of regulatory motifs in the upstream regions of *Glu* genes. The identification of specific cis-regulatory elements, such as ARBE and G-box, with higher frequencies in certain *Glu* genes suggests their putative involvement in specific biological processes.

Tissue expression results suggested that the berries exhibited a broad spectrum of *Glu* gene expression, with approximately 45 *Glu* genes being expressed in this tissue. However, the genes *LbaGlu16*, *LbaGlu34*, *LbaGlu32*, *LbaGlu40*, *LbaGlu09*, and *LbaGlu15*, which were expressed in the flowers and leaves of wolfberry, showed no expression in the berries. This suggests a tissue-specific regulation of these genes, possibly indicating distinct roles in flower and leaf development rather than in berry development. Additionally, the gene *LbaGlu39* demonstrated exclusive expression in flowers, hinting at its potentially pivotal role in flower growth and development. Conversely, genes such as *LbaGlu27*, *LbaGlu23*, and *LbaGlu43* exhibited expression solely in the leaves. The differential expression and enzymatic activity patterns of *LbaGlu28* between fertile and male-sterile cultivars suggest its potential role in callose degradation, anther development, and pollen viability. Furthermore, subcellular localization analysis confirmed the localization of *LbaGlu28* protein to the cell wall, supporting its functional role in the degradation of callose, a key component of the cell wall. Callose production, deposition, and subsequent degradation in anthers play crucial roles in pollen development in plants, and disruption of this process can lead to plant sterility [23,24]. Wan et al. identified a *Glu* gene mutation (*Osg1*) that disrupts callose degradation around the microspores in the anther locules [24]. However, further investigation is needed to fully understand the function of *LbaGlu28* in callose degradation and pollen development in wolfberry.

## 4. Materials and Methods

### 4.1. Plant Materials

A 7-year-old *L. barbarum* inbred line, Ningqi No. 1 (NQ1, fertile), and its natural mutant, Ningqi No. 5 (NQ5, sterile), were planted in Yuxin *L. barbarum* Ciyuan, Ningxia, China (106°4′56″ E, 38°29′46″ N), with a row spacing of 1.5 m and a plant spacing of 0.5 m and under routine management. The test site has an altitude of 1084 m, a mid-temperate continental climate, an average annual temperature of 8.5 °C, an average annual precipitation of 203 mm, and an average annual relative humidity of 60%.

During the germination period of flower buds from 2020 to 2021, 15 individuals of sterile NQ5 and fertile lines NQ1, respectively, were randomly collected for the cytological observation of anther development. The development of microspores was divided into five stages according to the length of the flower buds: archesporial cell stage (Ar, ≤1.3 mm), sporogenous cell stage (Sp, 1.31–1.89 mm), pollen mother cell stage (Pm, 1.90–2.93 mm), tetrad stage (Te, 2.94–4.0 mm), and pollen grain stage (Po, 4.01–5.0 mm) [25]. The anthers at different developmental stages were quickly stripped, and sections were prepared for observation.

### 4.2. Identification and Characterization of Glu Genes

To comprehensively identify *Glu* genes in wolfberry, we utilized Hidden Markov Models (HMMs) representing the GH17 domain (pafm00332) obtained from the Pfam database (https://pfam.xfam.org/, accessed on 8 January 2022) [26]. Employing HMMER 3.2, we conducted BLASTP alignments to detect potential *Glu* genes with default parameters. Subsequently, we verified the presence of the GH17 domain in each putative gene using batchCD (https://www.ncbi.nlm.nih.gov/Structure/bwrpsb/bwrpsb.cgi/, accessed on 8 January 2022), SMART (http://smart.embl-heidelberg.de/, accessed on 8 January 2022) [27], and the Conserved Domain (https://www.ncbi.nlm.nih.gov/cdd, accessed on 8 January 2022) databases [28]. Genes encoding proteins containing the GH17 domain were designated as *Glu* genes. Glu properties such as amino acid count, isoelectric point (pI), molecular weight (MW), and grand average of hydropathicity (GRAVY) were obtained from the ExPasy website (https://web.expasy.org/protparam/, accessed on 15 January 2022) [29]. Subcellular localization predictions for *Glu* genes were performed using WoLF PSORT (https://www.genscript.com/wolf-psort.html?src=leftbar, accessed on 15 January 2022) [30].

### 4.3. Relationship Between Chromosome Position and Replication of Glu Family Genes

The chromosomal positioning data, encompassing chromosome length and gene coordinates, were acquired from the Solanaceae Genomics Network (SGN) on 8 September 2022. TBtools (v1.045) was employed to construct a chromosomal distribution map delineating the arrangement of *Glu* genes [31]. Gene duplication events were scrutinized using Multiple Collinear Scanning Toolkits (MCScanX) with default parameters [32]. The collinear associations within the *Glu* gene family and their homologous counterparts in other species were visualized utilizing Dual Synteny Plotter software [31].

### 4.4. Multiple Sequence Alignment and Evolutionary Analysis

The *Glu* family members in *Arabidopsis thaliana* were identified employing a methodology analogous to that applied in the characterization of *Glu* family constituents in wolfberry. A cohort comprising 51 *Glu* family members from Arabidopsis was retrieved from public databases, and the sequences were subsequently analyzed using MEGA-X for evolutionary inference. This analytical endeavor encompassed the utilization of the neighbor-joining (NJ) algorithm and the JTT (Jones–Taylor–Thornton) protein evolution model, accompanied by the execution of 1000 bootstrap replicates and adherence to default parameters.

### 4.5. Gene Structure and Conserved Motif Analysis

The Gene Structure Display Server 2.0 (GSDS) (http://gsds.gao-lab.org/, accessed on 15 September 2022) was utilized to generate the gene structure map [33]. Conserved motifs within the *Glu* family genes were identified using MEME (https://meme-suite.org/meme/tools/meme, accessed on 15 September 2022). The analysis predicted a total of 10 motifs, with default parameters employed.

### 4.6. Prediction of Three-Dimensional Protein Structure of Wolfberry Glu

The SWISS-MODEL platform (https://swissmodel.expasy.org/, accessed on 7 October 2022) was employed for homology modeling to predict the three-dimensional structure of the protein [34,35]. Templates with a sequence identity exceeding 30% were chosen, and the protein model was subjected to evaluation using SAVES v6.0 (https://saves.mbi.ucla.edu/, accessed on 7 October 2022) [36]. Templates passing three or more assessments were selected as the final template. Visualization of the protein structure was conducted using the 3D protein structure visualization software VMD (http://www.ks.uiuc.edu/Research/vmd, accessed on 7 October 2022) [37].

### 4.7. Analysis of Cis-Acting Elements of the Glu Gene Family Members

The 2000 bp sequence upstream of the transcription initiation site was extracted as the promoter sequence of the *Glu* gene, and Plant CARE (http://bioinformatics.psb.ugent.be/webtools/plantcare/html/, accessed on 6 October 2022) was employed for the prediction of potential cis-acting elements on the promoter sequence [37]. The prediction outcomes were visualized, categorized, and analyzed.

### 4.8. Analysis of Glu Gene Expression in Different Tissues of Wolfberry

To explore variations in *Glu* gene expression across different tissues of wolfberry, FPKM (fragments per million exons mapped) data for the wolfberry variety Heinz 1706 with TFGD accession number D004 were utilized [38]. Gene expression levels were quantified by calculating the total FPKM values for each gene, taking into account gene length and read count. The data were normalized and visualized based on the average FPKM value for each gene. Additionally, the Euclidean distance between genes was analyzed, and hierarchical clustering was performed. The R package (v3.3) pheatmap was employed to generate the heatmap.

### 4.9. Observation of Sporopollenin Staining and Sporopollenin Enzyme Activity Assay

The anthers were initially rinsed twice with absolute ethanol for 1 min each time. Subsequently, they were equilibrated in 50% ethanol for 30 min, followed by removal and air-drying. The anthers were then immersed in PBS solution for 30 min to achieve equilibrium. Prior to use, a staining solution of safranin blue was prepared and applied to the anthers, followed by 1 h of light-shielded staining at room temperature. Subsequently, phenol fuchsin staining solution was dripped along one edge of the coverslip, with excess safranin blue solution absorbed from the opposite edge using filter paper until complete immersion, followed by a 15-min staining period. After staining, anthers were washed with PBS buffer and placed on a fluorescence microscope for observation and photographic documentation. Under fluorescence microscopy, cellular components stained red represent nuclear material, while yellow staining indicates sporopollenin. For the determination of β-1,3-glucanase activity, the assay was conducted according to the instructions provided with the assay kit. Briefly, 0.1 g of anthers at various developmental stages were weighed and homogenized in 1 mL of extraction buffer kept on ice. The homogenate was centrifuged at 12,000 rpm, 4 °C for 10 min, and the supernatant was collected and kept on ice. The absorbance was measured at 540 nm using an enzyme spectrophotometer.

### 4.10. Quantitative Real-Time Polymerase Chain Reaction (qRT-PCR)

Total RNA was extracted using the TRIzol method, with all materials RNase-free. Approximately 50 mg of tissue was homogenized in 1 mL Trizol. After centrifugation, the RNA-containing upper phase was mixed with isopropanol and incubated. The RNA pellet was washed with ethanol and dissolved in DEPC-treated water. Reverse transcription used the Hifair^®^ III 1st Strand cDNA Synthesis SuperMix (Yeasen Biotechnology, Shanghai, China). qRT-PCR included cDNA, Hieff^®^ qPCR SYBR Green Master Mix, forward and reverse primers, and ddH_2_O. The PCR program was as follows: step 1 (pre-denaturation), 95 °C, 30 s; step 2, 95 °C, 5 s; step 3, 60 °C, 30 s; repeated 40 cycles in total. Primer Premier 6.0 was used for all primer designs (Table S2). Relative expression levels were calculated using the 2-ΔΔCt method with actin as the reference gene. Triplicate experiments were performed, each with three technical replicates.

### 4.11. LbaGlu28 Cloning and Subcellular Localization

Based on the transcript information of *LbaGlu28* obtained from the transcriptome data (https://ngdc.cncb.ac.cn/gsa, GSA: CRA016215), primers were designed at the 5′ and 3′ ends, and PCR amplification was performed using genomic DNA (gDNA) as a template. The PCR amplification system consisted of 10 μL Taq enzyme Mix, 0.5 μL each of forward and reverse primers, 2 μL DNA template, and ddH2O to a final volume of 20 μL. The amplification conditions were as follows: initial denaturation at 95 °C for 3 min, followed by 35 cycles of denaturation at 95 °C for 10 s, annealing at 60 °C for 30 s, and extension at 72 °C for 15 s. Following the PCR experiment, 10 μL of the amplification product was subjected to 1% agarose gel electrophoresis. The PCR product was recovered using a gel recovery kit, ligated to the pGM-T vector using T4 DNA ligase, and transformed into *Escherichia coli* DH5α competent cells. Recombinant plasmids were screened by blue-white colony selection. Positive clones were confirmed by PCR using *LbaGlu28* primers to verify the presence of the target fragment. After the identification of positive clones, the recombinant plasmids were sent to Shanghai Sangon Biotech Co., Ltd. (Shanghai, China) for sequencing. Subcellular localization was conducted as follows: initially, tobacco seeds were sown, and after the emergence of cotyledons, approximately one month later, the experiment was conducted. The constructed vector plasmids were transformed into *Agrobacterium tumefaciens* (GV3101) using electroporation. The Agrobacterium was inoculated into 10 mL of YEB liquid medium and cultured at 170 rpm for 1 h. The culture was then centrifuged at 4000 rpm for 4 min, the supernatant was discarded, and the bacterial pellet was collected. The bacterial pellet was resuspended in 10 mM MgCl_2_ suspension to adjust the OD600 to approximately 0.6. Subsequently, healthy tobacco plants were selected, and the plasmids were injected into the lower epidermis of tobacco leaves using a needleless syringe, with proper labeling. The injected tobacco plants were cultured under weak light conditions for 2 days. Afterward, the injected tobacco leaves were harvested, processed into slides, and observed using confocal laser scanning microscopy, with photographic documentation performed.

### 4.12. Statistical Analysis

All analyses were conducted using Prism GraphPad 7.0 software (https://www.graphpad.com, accessed on 20 Noverber 2022). All variables were shown as the mean ± standard deviation (SD). The Student’s *t*-test and ANOVA were applied for data analysis. *p* < 0.05 was considered statistically significant.

## 5. Conclusions

Overall, the findings presented in this study contribute to our understanding of the molecular mechanisms underlying *Glu* gene function and regulation in wolfberry. Further research exploring the functional characterization of *Glu* genes and their potential roles in agronomic traits, such as reproductive development and stress responses, will enhance the utilization of these genes for crop improvement strategies in wolfberry and related species.

## Data Availability

The datasets supporting our conclusions of the current study are included in the manuscript and additional file. The Arabidopsis DREB protein sequences were downloaded from the Arabidopsis information source database (https://www.arabidopsis.org, accessed on 7 October 2022). The original transcriptome data reported in this paper has been stored in the genome sequence archive (Genomics, Proteomics & Bioinformatics 2021) of the National Genome Data Center (Nucleic Acid Research 2022) of China National Bioinformatics Center/Chinese Academy of Sciences Beijing Genome Research Institute (GSA: CRA016215), and can be downloaded from https://ngdc.cncb.ac.cn/gsa.

## Supplementary Materials

The following supporting information can be downloaded at https://www.mdpi.com/article/10.3390/plants14010052/s1, Figure S1: GH17 domain in *Glu* genes; Figure S2: X8 domain in *Glu* genes; Table S1: The information of *Glu* gene family in wolfberry; Table S2: qRT-PCR primers.
