# Supplementary material for: Comprehensive Analysis of β-1,3-Glucanase Genes in Wolfberry and Their Implications in Pollen Development
Source: Plants (Basel). 2024 Dec 27;14(1):52. doi: 10.3390/plants14010052 (PMC11722940; doi:10.3390/plants14010052)
Supplement: Supplementary file 1 [file plants-14-00052-s001.zip › Supplementary File S1(DNAMAN-GH17).pdf]

```
LbaGlu02   ........................................   0
LbaGlu03   ........................................   0
LbaGlu04   ........................................   0
LbaGlu07   ........................................   0
LbaGlu09   MDHLNPSKHALTPIVLASKQSPTINSHSLLHSLTHPICLW  40
LbaGlu10   ........................................   0
LbaGlu13   ........................................   0
LbaGlu14   ........................................   0
LbaGlu19   ........................................   0
LbaGlu28   ........................................   0
LbaGlu32   ........................................   0
LbaGlu33   ........................................   0
LbaGlu34   ........................................   0
LbaGlu35   ........................................   0
LbaGlu38   ........................................   0
LbaGlu39   ........................................   0
LbaGlu40   ........................................   0
LbaGlu42   ........................................   0
LbaGlu43   ...........................MEPVSNTPLSCAL  13
LbaGlu44   ........................................   0
LbaGlu45   ........................................   0
LbaGlu58   ........................................   0
Consensus

LbaGlu02   ..................MMTTLSVFVFAGMLILTYLEII  22
LbaGlu03   ................MAILFFNFKALLLLLSLTDLFAST  24
LbaGlu04   .................MANSSFIFLWFILLLNGIIAVHG  23
LbaGlu07   ..........MNEFCFNWVVSISFMLLLIFTNNAQPTARA  30
LbaGlu09   LPLALASFSSTSTMASINPIHCFSSTIVLIVVIIVFLSFV  80
LbaGlu10   ....................MKMVLSFFILSLLMFPSSST  20
LbaGlu13   ..............MASTKPHFFVTAVLIVVLILATANFT  26
LbaGlu14   ..............MACTKPHFFVTAVLIVLLILATSDFT  26
LbaGlu19   ..............MASTKSQFSFSLILFGVFTLATLDFT  26
LbaGlu28   ................MGKIIQEVLFLLYCVFILLGNYST  24
LbaGlu32   ...........MVFCSKNGLFPAASILIVGLLMCSTQMKG  29
LbaGlu33   ...........MATSDKYTTPQMAAIILLGLIVANN.DIA  28
LbaGlu34   ...........MAFCSKNGLFPAASILIVGLLMCSTQMKG  29
LbaGlu35   ..........MDAPLRSRTSLWQILSTFFLLILLGLNNIG  30
LbaGlu38   ......MLYYFYLTFPKKMTSSLSLLLCLILLHNVIVAGG  34
LbaGlu39   ...........MAFCSKNGLFPAASILIVGLLMCSTQMKG  29
LbaGlu40   ..........MATSSCKHTTTQMAAIMLLGLLFASSIDIA  30
LbaGlu42   .........MALCYLFTKKGFAAAVLLLFGLLMSSDQITG  31
LbaGlu43   GALIVLLAPIFLQGKCVTHYFNNTCFIVSVHNLNFSPVIG  53
LbaGlu44   ......................MQRLCVLLFVFACVTLSS  18
LbaGlu45   .......................MMESLTLLLACIFLSPV  17
LbaGlu58   .................MANLSVFVFVFVGVLILTCLQII  23
Consensus

LbaGlu02   EALSVGVCYGRNGNNLPSAQIVVNLYKANG.ITNMPVYDP  61
LbaGlu03   LSLGIGINYGQIANNLPTPSPVSTLLRSLN.VTPVKLYDA  63
LbaGlu04   MKGTYGINYGKISDNIPTPENVLIILKKNK.IKNIRIYDA  62
LbaGlu07   FTGTYGINYGRIADNIPSPDKVVKLLRAAK.IKNVRIYDA  69
LbaGlu09   MATSIGVNYGQIADNLPPPEKVVPIVKSMG.ATPVKLYDA 119
LbaGlu10   STTTIGITYSPGIN.SPPPEHVVTALQSLK.ISSVRLLNP  58
LbaGlu13   GAQT.GVCYGRNGNGLPSPAIVVALCNRNN.IRRMRIYDP  64
LbaGlu14   GAQT.GVCYGRIGNGLPSPAIVVALCNQNN.IRRMRIYDP  64
LbaGlu19   GAQT.GVCYGRLGNGLPSPAIVVSLCNRNN.IRRMRIYDP  64
LbaGlu28   VEASIGVCYGPVGTNLPPISEAINLIKSNG.ISRIRLFNP  63
LbaGlu32   V.QSVGVCYGKIANNLPSEQIVIKLYNANG.IKKMRIYYP  67
LbaGlu33   GAQSIGVCYGMLGNNLPPASQVIQLYKSRN.IRRMRLYDP  67
LbaGlu34   V.QSVGVCYGKIANNLPSEQIVIKLYNANG.INKMRIYYP  67
LbaGlu35   GVESLGINYGQVANNLPPPDKVLQLLRALK.ITKTRIYDT  69
LbaGlu38   FTGTYGINYGRIADNIPAPESVVTLLQANK.IKNVRIYDA  73
LbaGlu39   V.QSVGVCYGKIANNLPSEQIVINYTMLMA.LKKMRIYYP  67
LbaGlu40   GAQSIGVCYGMMGNNLPNHQPVIQLYKSRN.IGRLRLYDP  69
LbaGlu42   AYSNIGVCYGKIANNLPSDQIVINLYKANG.IRKMRIYSP  70
LbaGlu43   VEGNIGVNYGTVADDLPPPVQVARPLLESTPIRPVRLFDA  93
LbaGlu44   GVEGIGVNYGLLGNNLPPPAQVINLLKSKN.IQNIRIFDP  57
LbaGlu45   IVASLGINYGQIADNLPSPENVVPIVKSIG.ATRIKLYDA  56
LbaGlu58   EAQLIGVCYGINGNNLPLPIIVVDLYKANG.ITKMRPYDP  62
Consensus       g  y       p
```

```
LbaGlu02    IPETLITALKESNIGIILDIPNENLQALTD..PKAATDWVN    99
LbaGlu03    DPNVLTAFANTNVEFVIGLGNENLQRMS.D.PQQAQSWIQ    101
LbaGlu04    DQPVLKAFSGSGIEISVCLPNELLIDVSKN.GSIALEWIQ    101
LbaGlu07    EPSVLNAFKGTGLEIVIGLPNGIVKEMGAN.ADHALNWVK    108
LbaGlu09    DPHVLKAFANSGVEFIVSLGNEHLSNMK.D.PAKAQVWVK    157
LbaGlu10    TPSLIRAFSYSNISLLLTVPNHIVTSFASN.RSSATLWLY     97
LbaGlu13    QQDTLQALRGSNIELMLGVPNPDLRNIASS.QANANTWVQ    103
LbaGlu14    QQDTLQALRGSNIELMLGVPNPDLQNIASS.QANADTWVQ    103
LbaGlu19    HQPTLQALRGSNIEVTLGVPNTDLENVAAS.QANANTWIQ    103
LbaGlu28    DPEALQPFCGTGIELLIGVPNEILPTLANNEVTTSIEWLQ    103
LbaGlu32    DKNVFNALKGSNIEIILIVPNQDLEALTN..PSSANAWVQ    105
LbaGlu33    NQAALQALRGSNIEVMLGVPNSDLQNLAAN.PSNANNWVQ    106
LbaGlu34    DKNVFNALKGSNIEIILIVPNQDLEALTN..PSSANAWVQ    105
LbaGlu35    NPQVLTTFANSNIELIVTVENQMLATLC.D.QQQALQWVT    107
LbaGlu38    DKGVLTAFKGSGIQIIIGLGNEFLKDISVN.EDRAVEWVK    112
LbaGlu39    DKNVFNALKGSNIEIILIVPNQDLEALTN..PSSANAWVQ    105
LbaGlu40    NHGALQALKGSNIEVILGLPNSIVKHIASG.IEHAKWWVQ    108
LbaGlu42    VTNIFNALKGSSIEILLIVPNQDLEALAN..SSKANTWVQ    108
LbaGlu43    NPEILQAFAHTDIAVTINVPNDLIPHLTK..LSIAQQWVE    131
LbaGlu44    NQDVLKALEGSGISIILGTRNEDLQALASD.PTFATNWVV     96
LbaGlu45    DPKVLKAFANTGVEFIVSLGNEYLAKMK.D.PSNAQSWVK     94
LbaGlu58    IPEMLIALKGSNIEIVLDIPNDKLQALND..PKEATNWVI    100
Consensus                        n             w

LbaGlu02    ANIMSYSSNVKFKYISVGNEISPGNVAKAKFAPFL.....    134
LbaGlu03    QHVQPYHTQTKITCIIVGNEVLTG.DDMQLKSYLL.....    135
LbaGlu04    VNLQPFLPGTSIRGIAVGNEILGG..DTAIAEAIV.....    134
LbaGlu07    DNVKAFLPETRIVGIAVGNEVLGG.SDNELEVALL.....    142
LbaGlu09    TNVQAYLPATKITCIAVGNEVLTF.NDSSLSDNLL.....    191
LbaGlu10    NNVLPFHPRAKISLISVGSIVISSVVSPDPSIVIV.....    132
LbaGlu13    NNVKNY.GNVRFRYIAVGNEISPLNGNTQQYVPFL.....    137
LbaGlu14    NNVKNY.GNVRFRYIAVGNEISPLNGDTQQYVPFL.....    137
LbaGlu19    NNVKNY.GNVKFRYIAVGNEVSPLNENSK.YVPVL.....    136
LbaGlu28    SNIFAHVSPNQVKYIVVGNEIFLK...DPYYSPYI.....    135
LbaGlu32    DNVRSYIPEVKFKYIAVGNEVDPG.TNTGQYAQYV.....    139
LbaGlu33    RNVRNFWPAVKFRYIAVGNEVSEVTGTSS.FTRYL.....    140
LbaGlu34    DNVRSYIPYVKFKYIAVGNEVDPG.TNTGQYAQYV.....    139
LbaGlu35    SHIRPYFPATNITGIAVGNEIFTD.GDTSLMTYIV.....    141
LbaGlu38    INVQPYLPDTLISGIAVGNEILGG.GIVEIWEAIV.....    146
LbaGlu39    DNVRSYIPDVKFKYIAVGNEVDPG.TNTGQYAQYVCRDNV    144
LbaGlu40    KNVKDFWPDVKIKYIAVGNEISPVTGTSY.LTSFL.....    142
LbaGlu42    DNIKNYFPDVKFKYIAVGNEVDPG.TNTGQYARFV.....    142
LbaGlu43    LNVLPYVPATNIVRIIVGNEVIST.ANKLLIVSIV.....    165
LbaGlu44    TNIIPHTSNVKFTCISAGNEVIPG....PLSTFVL.....    127
LbaGlu45    KNVQAYLPATKITCIAVGNEILTF.NDSTLTDNLL.....    128
LbaGlu58    VNVMNYFSQVKFKYINVGNEVSPINVATSQFAPFL.....    135
Consensus                     g

LbaGlu02    LPALQNVQQAITTFHLQDQVKVTTSIETGMLTSTYPPSHS    174
LbaGlu03    .PAMQGVYKAIVNLGLSNDIYVAHPHSAGILENSFPPSSG    174
LbaGlu04    .PAVKSVYRALRKLGLTDTIEVSTPHSEAVFNSTYPPSDG    173
LbaGlu07    .NAVKNVYNATKKLGISDIVQLSTAHSQAVFADSFPPSFC    181
LbaGlu09    .PAMESVYAAIVNCNLDKQVSVTTAHSVAILETSYPPSAG    230
LbaGlu10    .PAMQNLHHALLDLGIRTVSVSTTFSFINVITTAFPPSSA    171
LbaGlu13    LNAMRNIQNAISGAGLGNQIKVSTAIETGLTTDTFPPSKG    177
LbaGlu14    LPAMRNIQNAISGAGLRNQIKVSTAIETGLTTDTSPPSKG    177
LbaGlu19    LNAIRNIQTAISGAGLGNQIKVSTAIETNLTTDTSPPSNG    176
LbaGlu28    VPIITKLYQALQTLGLATTIKLSSSHASTILSNSYPPSSS    175
LbaGlu32    APAMENVHNALSAAGLQDQIKVSTATYSGLLANTYPPKDG    179
LbaGlu33    LPAMRNIQNAISSAGLRNNIKVSTSVDMTLIGNSFPPSQG    180
LbaGlu34    APAMENVHNALSAAGLQDQIKVSTATYSGLLINTYPPKDG    179
LbaGlu35    .PAMVNLHAAIVKTGLSQSIQVSSPNSLAVLANSYPPSSG    180
LbaGlu38    .PAAKNVYSALDKLDLAHKIEVSSPHSEAVFEDTYPPSAG    185
LbaGlu39    AEVMENVRNALSAAGLQDKIKVSTATYSGLLANTYPPKDG    184
LbaGlu40    IPAMVNIYRAVGEAGLGNNVKVSTSVDMTLIGNSYPPSQG    182
LbaGlu42    GPAMGNIYNVLSAAGLQDKIKVSTATYSGLLANTYPPKDS    182
LbaGlu43    .PAMETLHAAIVEKSLDRHIQISTPHSLGILTNSSPPSTG    204
LbaGlu44    .GAMQNLDSALKANNL..NIPVSTAVPLQVLGTSYPPSNG    164
LbaGlu45    .PAMQSVHTAIVNLKLDKQVIVTTAHSLAILETSYPPSAG    167
LbaGlu58    LSAMTNVQQAITKFKLQNQVKVTTAIETGLLANTYPPSQS    175
Consensus                                  pp
```

```
LbaGlu02    VFRNDA.ISFIKPIIEFLKQNQSPLLANIYPYYGYKANPD    213
LbaGlu03    SFKQDL.SDYIRGMLNFHAQTKSPFLINVYPFFAYKDNPN    213
LbaGlu04    AFKPSL.MPYLGPLLQFFNFVGSPFYINAYPFLAYKFEP.    211
LbaGlu07    VFKDGV.AELMKPLLDFFSKIGSPFCLNAYPFLAYTYNSD    220
LbaGlu09    AFRRDL.VKCITQVLDFHCKTGSPFLINAYPYFAYKADPK    269
LbaGlu10    EFQDPVNKIVISPVLEFLEETNSSMLMNVYPYNVYQLHG.    210
LbaGlu13    KFRDIV.RQFIDPIISFIVANRSPLIVNIYPYFAIANNR.    215
LbaGlu14    KFRDIV.RRFIDPIISFIVENRSPLIVNIYPYFAIANNR.    215
LbaGlu19    RFKDIV.RRFIQPIIDFLKTNSAPLIVNLYPYFAVANNP.    214
LbaGlu28    TFDSNI.KPFLIPLLQFLHDTRSPLMVNVYPFFAYINNPK    214
LbaGlu32    IFREEY.KNFINPIIGFLAQNNLPLLANIYPYFSHTDNPA    218
LbaGlu33    SFRNIV.RSFIDPIIGFLRGINSPLIVNIYPYFSYSGNPR    219
LbaGlu34    IFREEY.KNFINPIIGFLAQNNLPLLANIYPYFGHTDNPA    218
LbaGlu35    SFRTDL.NGIMQQFLQFLSTTRSPFWINAYPYFAYKDSPT    219
LbaGlu38    VFKQSI.LPYMLPLLNFSSQIGSPFYINAYPFLAYKSDPS    224
LbaGlu39    IFREEY.KNFINPIIVFLAQNNLPLLANIYPYFGHIDNPA    223
LbaGlu40    SFRNDA.RWFIDPIVGFLRDTRAPLIVNIYPYFSYSGNPG    221
LbaGlu42    NFREEF.KGFINPIIEFLARNNLPILANLYPYFGHIYNTV    221
LbaGlu43    KFRAGYDTHVLKPLLEFLRATNSPFMINPYPFFGSSDNT.    243
LbaGlu44    AFSEDS.IQFLKPIAQFLATKKYPLLANVYPYFAYSGNSA    203
LbaGlu45    AFRKDL.VDCVTQIVDFHCKTGSPFLINAYPYFAYKGNPK    206
LbaGlu58    VFRDIV.SSFINSIIAFLKQNNSPLLANIYPYFAYIGDPD    214
Consensus    f              f           n yp

LbaGlu02    RIPLPYALFTQQK.......PDPSGYQNLFDAMLDAVYYA    246
LbaGlu03    EVPLDYVLFRPNQGTI..DPATNLKYDNMLYAQIDAVYSA    251
LbaGlu04    TIDINYALFKKNKGIV..DPKTKLHYDNMFDAMVDATYIA    249
LbaGlu07    KIDINYALFQPNEGIV..DNKTNLHYDNLLDAQIDAAYAA    258
LbaGlu09    QVPLDFVLFQSNQGIL..DPATNLRYDNMLFAQIDAVHSA    307
LbaGlu10    EIPVGFALFEETPFNFRDIVVTGVRYHNLFDMMVDSVIAA    250
LbaGlu13    EIKLEYALFTSPGVVV..N.DNGKGYRNLFDALLDATYSA    252
LbaGlu14    DIKLEFALFTSPGVVV..N.DNGKGYRNLFDALLDATYSA    252
LbaGlu19    EINLEYALFTSPIVVV..N.DNGRGYRNLFDAILDATYSA    251
LbaGlu28    YVSLDHALFRSSY..V..EYDQNLDYDNMFDASIDAFVYA    250
LbaGlu32    NIPLSYALFNQQE.......KNDAGYQNLFDALLDSMYFA    251
LbaGlu33    DISLPYALFTAPNVVV..Q.DGSLGYRNLFDAMLDAVYAA    256
LbaGlu34    KIPLSYALFNQQE.......KNDAGYQNLFDALLDSMYFA    251
LbaGlu35    KISLDYVLFNSNQGMV..DPYTKLHYDNMLYAQVDAAISA    257
LbaGlu38    HIDLNYALFEKSTGIY..DAKTKLHYDNMFEAMIDAAYFA    262
LbaGlu39    DILFSYALFNQQE.......KNDAGYQNLFDALLDSMYFA    256
LbaGlu40    QISLPYALFTAPNVVV..Q.DGSRQYRNLFDAMLDSVYAA    258
LbaGlu42    IVPLSYALFNQQG.......TNSIGYQNLFDALLDSFYFA    254
LbaGlu43    ...LDYALFRPNPGVF..DETTRLTYTNMLDAQLDAIFSA    278
LbaGlu44    QIQLDYALLKNTAPIS..S.DGQFQYNNMFDAMVDSLYAA    240
LbaGlu45    QVSLDFVLFQPNSGIV..DPESNLHYDNMLFAQIDAVHSA    244
LbaGlu58    HVALPYALFTQQE.......PDPSGYYNLFDAMLDSIYYA    247
Consensus          l                 y n      d    a

LbaGlu02    VEKAGGDNIEIVVAESGWPSDGGG..VGVSMDNAATYYRN    284
LbaGlu03    IKAMGYSDIPVKVSETGWPSKGDQNEFGATRENAALYNGN    291
LbaGlu04    LEKLGYKKMCVIVSETGWASKGDDNEAGATPENARTYNFN    289
LbaGlu07    LEDAGFRKMEVIVTETGWASNGDENEPAATPGNARTYNYN    298
LbaGlu09    LASIGYKNVCVQISETGWPSKGDADELGATLDNARKYNCN    347
LbaGlu10    MAVSGYENVPLILTETGWPSNDD...DETTKMYAEKYLQG    287
LbaGlu13    LEKAGGSSLEIVVSESGWPSAGAG..QLTSIDNARTYNNN    290
LbaGlu14    LEKAGGSSLEIVVSESGWPSAGAG..QLTSIDNARTYNNN    290
LbaGlu19    LEKAGGSSLQIVVSESGWPSAGAG..QLTSIENARTYNNN    289
LbaGlu28    MEKEGFEGIPVMVTETGWPTAGI...DGASIDNAFTYNEN    287
LbaGlu32    TEKLGGQNIEIIVSESGWPSEGD...PAATIENAQTYYTN    288
LbaGlu33    LSRAGGGSIEIVVSESGWPSAGAF..GATT.NNAATYYRN    293
LbaGlu34    TEKLGGQNIEIIVSESGWPSEGD...PAATIENAQTYYTN    288
LbaGlu35    IARMGFNGLEVKISETGWPSKGDSNEIGATLQNAAIYNRN    297
LbaGlu38    LENAGFEKMPVICSETGWASNGDENEAGANVKNARTYNKN    302
LbaGlu39    TEKLGGQNIEIIVSESGWPSEGD...PAATIENAQTYYTN    293
LbaGlu40    MDRSGGGSVGIVVSESGWPSAGAF..GATT.DNAATYLRN    295
LbaGlu42    IEKARGPNVEIVVSESGWPAEGN...PSATIENAQTYYRN    291
LbaGlu43    LKLLDFEDIEIVIAETGWPSIGDPGQAGVDAGSAAEYNRK    318
LbaGlu44    LEKVGQPAVIVVVTETGWPSAGD...VYATKDNAQTYANN    277
LbaGlu45    LASVGYKNVCVQISETGWPSKGDEDEAGATAENARKYNCN    284
LbaGlu58    TEKAGGDNVEIVVSESGWPSDGG...VGASIDNAATYYRN    284
Consensus                  e gw               a  y
```

```
LbaGlu02    LISHVKSTSGTIHKPGKAIETYLFAMFDENVKIGGETEKH    324
LbaGlu03    LFQRIQENQGTPANPSEPIIVYVFALFNENLKPGPGSERN    331
LbaGlu04    LHKRLMKKKGTPYKPKTKAKAYVFALFNENLKPGPTSERN    329
LbaGlu07    LRKRLAKRKGTPMRPKKMLKAYIFALFNEYQKPGQSSEKN    338
LbaGlu09    LIKIVSQKKGTPLKPNNNLNIYVFALFNENLKPGPTSERN    387
LbaGlu10    LISHLKSGLGTPLRKQGVAEAYIYQLFDDTNNLTSNTQGG    327
LbaGlu13    LIKHVKE..GSPKRPSRPIETYIFALFDEDQK.SPELEKH    327
LbaGlu14    LIKHVKE..GSPKRPSRPIETYIFDLFNEDRK.SPEFEKH    327
LbaGlu19    LIQHVKG..GSPKRPSGPIETYVFALFDEDQKKNGEIEKH    327
LbaGlu28    IVRKALNNVGTPKRPGVGLDIFLFDLFDENGKSGEEFERH    327
LbaGlu32    LINHVKGGAGTPKKPGKTIETYLFAMFDENQKQGKISEKH    328
LbaGlu33    LIQHVKR..GSPRRPNFVIETYLFAMFDENNK.NPELEKH    330
LbaGlu34    LINHVKGGAGTPKKPGKTIETYLFAMFDENQKQGKISEKH    328
LbaGlu35    IFRRQLLNEGTPLRPNVRLDIYVFALFNEDMKPGPTSERN    337
LbaGlu38    LRKKLLKKKGTPYRPKMVMRAYVFALFNENLKPGPTSERN    342
LbaGlu39    LINHVKGGAGTPKKPGKTIETYLFAMFDENQKQGKISEKH    333
LbaGlu40    LIQHAKE..GSPRKPG.PIETYIFAMFDENNK.NPELEKH    331
LbaGlu42    IVNHVKSRAGTPKKPGRIIETYLFAMFDENQKEGAVTEQH    331
LbaGlu43    LIQHVMSGIGTPLMPNRTFETYIFALFNEDLKPGPTFERN    358
LbaGlu44    LIAHVSSGQGTPRRPGKVLETYIFALFNENQK.PAGTEEN    316
LbaGlu45    LMKLISQKKGTPMKPNNDLNIFVFALFNENQKPGPGSERN    324
LbaGlu58    LIRHVKSGAKTIHKPGNAINAYLFAMFDENEKPGAETEKH    324
Consensus                              f

LbaGlu02    FGVFYPDKTQKYNLTF........................    340
LbaGlu03    YGLYYPDGTAVYNIGLQQGFLPRMDYSAATKNVLSSSAFL    371
LbaGlu04    FGLFKADGSIAYDIGFKGIVSSSPSSKDFVLRGRFWISQS    369
LbaGlu07    FGLYKADGSISYDIGFPGLQDISAASSLLSLKGIQAQGYY    378
LbaGlu09    YGLFKPDGSPSYPLGFSGINAGGSTNSSSGSSPKAAGSGS    427
LbaGlu10    EPVQYWGVMYSSNMTMKYNINFNNAHHNSSMIPLIVVPSI    367
LbaGlu13    FGLFSPNRQPKYPISFN.......................    344
LbaGlu14    FGLFTPNRQPKYSISFN.......................    344
LbaGlu19    FGLFSANMQPKYQISFN.......................    344
LbaGlu28    FGIFGDNGIKAYDIRFN.......................    344
LbaGlu32    FGLYYPDQRPKYQLNFN.......................    345
LbaGlu33    FGLFFPNKQPKYNLNFGVSDFVWDISAETNATASLISEM.    369
LbaGlu34    FGLFYPDQRPKYQLNFN.......................    345
LbaGlu35    YGLFQPDGTMVYNVGLLSTTSTTTEPSSASISLASSAPPM    377
LbaGlu38    FGLFKADGSIAYKIGFRGIVPSS.ASKDFVLRGGFWSFQW    381
LbaGlu39    FGLFYPDQRPKYQLNFN.......................    350
LbaGlu40    FGLFSPNKQPKYNLNFGVSDFVWDISAETNTTASLISEM.    370
LbaGlu42    FGLFYPNQTAKYDLKFMYSD....................    351
LbaGlu43    FGLFKPDMTEVYDIGILRPMVINAADAAAETDHSRTNSCT    398
LbaGlu44    FGLFYPDMTEVYHVNLTP......................    334
LbaGlu45    YGLFKPDGSQAYPLGVPAVTLGTSNTTSATS.....GSPT    359
LbaGlu58    FGVFHPDKTQKYNLTF........................    340
Consensus

LbaGlu02    ........................................    340
LbaGlu03    LLFVILF.................................    378
LbaGlu04    FIIAACATIIVLS...........................    382
LbaGlu07    LPAIAITTSISVLLSRL.......................    395
LbaGlu09    SSTPTTWSPQDGSSSSGYMSITSDSGR.VLFCWKSLILQL    466
LbaGlu10    YFLYHLLWLFWIVD..........................    381
LbaGlu13    ........................................    344
LbaGlu14    ........................................    344
LbaGlu19    ........................................    344
LbaGlu28    ........................................    344
LbaGlu32    ........................................    345
LbaGlu33    ........................................    369
LbaGlu34    ........................................    345
LbaGlu35    VKQVGYQSLANWMMMFAYFCWLLMR...............    402
LbaGlu38    FIVVASIVMLLQFVDL........................    397
LbaGlu39    ........................................    350
LbaGlu40    ........................................    370
LbaGlu42    ........................................    351
LbaGlu43    NLLMLLIVCLTCL...........................    411
LbaGlu44    ........................................    334
LbaGlu45    ATTPSASLPPASSSS.GYLAITSDSGRSPAHCKCSLLYIV    398
LbaGlu58    ........................................    340
Consensus
```

```
LbaGlu02    ............                                  340
LbaGlu03    ............                                  378
LbaGlu04    ............                                  382
LbaGlu07    ............                                  395
LbaGlu09    HIIGIVSLLFSQ                                  478
LbaGlu10    ............                                  381
LbaGlu13    ............                                  344
LbaGlu14    ............                                  344
LbaGlu19    ............                                  344
LbaGlu28    ............                                  344
LbaGlu32    ............                                  345
LbaGlu33    ............                                  369
LbaGlu34    ............                                  345
LbaGlu35    ............                                  402
LbaGlu38    ............                                  397
LbaGlu39    ............                                  350
LbaGlu40    ............                                  370
LbaGlu42    ............                                  351
LbaGlu43    ............                                  411
LbaGlu44    ............                                  334
LbaGlu45    AFISIVLHLQC.                                  409
LbaGlu58    ............                                  340
Consensus
```

Fig S1 GH17 domain in *Glu* genes
