# Supplementary material for: Comprehensive Analysis of β-1,3-Glucanase Genes in Wolfberry and Their Implications in Pollen Development
Source: Plants (Basel). 2024 Dec 27;14(1):52. doi: 10.3390/plants14010052 (PMC11722940; doi:10.3390/plants14010052)
Supplement: Supplementary file 1 [file plants-14-00052-s001.zip › Supplementary File S2(DNAMAN-X8).pdf]

```
LbaGlu01    ........................................     0
LbaGlu05    ........................................     0
LbaGlu06    ........................................     0
LbaGlu08    ........................................     0
LbaGlu12    ........................................     0
LbaGlu15    MVQCQTKSNSPSVKIFCFHFVSLSLTFLSQFLFLHIFSFF    40
LbaGlu16    ........................................     0
LbaGlu17    ........................................     0
LbaGlu18    ........................................     0
LbaGlu20    ........................................     0
LbaGlu21    ........................................     0
LbaGlu22    ........................................     0
LbaGlu23    ........................................     0
LbaGlu24    ........................................     0
LbaGlu25    ........................................     0
LbaGlu26    ........................................     0
LbaGlu27    ........................................     0
LbaGlu29    ........................................     0
LbaGlu30    ........................................     0
LbaGlu31    ........................................     0
LbaGlu36    ........................................     0
LbaGlu37    ........................................     0
LbaGlu41    ........................................     0
LbaGlu46    ........................................     0
LbaGlu47    ........................................     0
LbaGlu48    ........................................     0
LbaGlu49    ........................................     0
LbaGlu50    ........................................     0
LbaGlu51    ........................................     0
LbaGlu52    ........................................     0
LbaGlu53    ........................................     0
LbaGlu54    ........................................     0
LbaGlu55    ........................................     0
LbaGlu56    ........................................     0
LbaGlu57    ........................................     0
Consensus
```

```
LbaGlu01    ........................................     0
LbaGlu05    ........................................     0
LbaGlu06    ........................................     0
LbaGlu08    ........................................     0
LbaGlu12    ........................................     0
LbaGlu15    LILIPFSIYQHLNSIVKIPLFFFFGSRYHFCYNKFQDSAY    80
LbaGlu16    ........................................     0
LbaGlu17    ........................................     0
LbaGlu18    ........................................     0
LbaGlu20    ........................................     0
LbaGlu21    ........................................     0
LbaGlu22    ........................................     0
LbaGlu23    ........................................     0
LbaGlu24    ........................................     0
LbaGlu25    ........................................     0
LbaGlu26    ........................................     0
LbaGlu27    ........................................     0
LbaGlu29    ........................................     0
LbaGlu30    ........................................     0
LbaGlu31    ........................................     0
LbaGlu36    ........................................     0
LbaGlu37    ........................................     0
LbaGlu41    ........................................     0
LbaGlu46    ........................................     0
LbaGlu47    ........................................     0
LbaGlu48    ........................................     0
LbaGlu49    ........................................     0
LbaGlu50    ........................................     0
LbaGlu51    ........................................     0
LbaGlu52    ........................................     0
LbaGlu53    ........................................     0
LbaGlu54    ........................................     0
LbaGlu55    ........................................     0
LbaGlu56    ........................................     0
LbaGlu57    ........................................     0
Consensus
```

```
LbaGlu01   ................................MATQKLLS     8
LbaGlu05   ........................................     0
LbaGlu06   ...............................MEKPNK...     6
LbaGlu08   .......................MSANIFSTTIMEHFNFR    17
LbaGlu12   ...............................MAILFY...     6
LbaGlu15   PSAKFIIFFNKNLTFSEKIETFSCENIGYFSFCIPLFFLW   120
LbaGlu16   ..............................MGCLSTL...     7
LbaGlu17   ........................................     0
LbaGlu18   ........................................     0
LbaGlu20   .......................................M     1
LbaGlu21   .............................MGNQKNNDFLI    11
LbaGlu22   .....................................MAH     3
LbaGlu23   ..................................MEIMSS     6
LbaGlu24   .......................................M     1
LbaGlu25   ..................................METNNA     6
LbaGlu26   ....................................MPMM     4
LbaGlu27   ......................................MA     2
LbaGlu29   .................................MEFFAFS     7
LbaGlu30   ...............................MEKLTI...     6
LbaGlu31   ........................................     0
LbaGlu36   ..........................MANTKKFPPFFFFV    14
LbaGlu37   .................................MILMTLK     7
LbaGlu41   ........................................     0
LbaGlu46   ...................................MSLLG     5
LbaGlu47   .................................MGILGMK     7
LbaGlu48   ........................................     0
LbaGlu49   ...............................MATFTF...     6
LbaGlu50   ..................................MARKYS     6
LbaGlu51   ......................................MG     2
LbaGlu52   .................................MYKIISS     7
LbaGlu53   ...............................MEVSNKKIT     9
LbaGlu54   ............................MMSGACCRLFLI    12
LbaGlu55   ..........................MKTLSLYDILLLIL    14
LbaGlu56   .................................MGLFSFS     7
LbaGlu57   ...............................MEMFIK..T     7
Consensus
```


```
LbaGlu01   ................................MATQKLLS     8
LbaGlu05   ........................................     0
LbaGlu06   ...............................MEKPNK...     6
LbaGlu08   .......................MSANIFSTTIMEHFNFR    17
LbaGlu12   ...............................MAILFY...     6
LbaGlu15   PSAKFIIFFNKNLTFSEKIETFSCENIGYFSFCIPLFFLW   120
LbaGlu16   ..............................MGCLSTL...     7
LbaGlu17   ........................................     0
LbaGlu18   ........................................     0
LbaGlu20   .......................................M     1
LbaGlu21   .............................MGNQKNNDFLI    11
LbaGlu22   .....................................MAH     3
LbaGlu23   ..................................MEIMSS     6
LbaGlu24   .......................................M     1
LbaGlu25   ..................................METNNA     6
LbaGlu26   ....................................MPMM     4
LbaGlu27   ......................................MA     2
LbaGlu29   .................................MEFFAFS     7
LbaGlu30   ...............................MEKLTI...     6
LbaGlu31   ........................................     0
LbaGlu36   ..........................MANTKKFPPFFFFV    14
LbaGlu37   .................................MILMTLK     7
LbaGlu41   ........................................     0
LbaGlu46   ...................................MSLLG     5
LbaGlu47   .................................MGILGMK     7
LbaGlu48   ........................................     0
LbaGlu49   ...............................MATFTF...     6
LbaGlu50   ..................................MARKYS     6
LbaGlu51   ......................................MG     2
LbaGlu52   .................................MYKIISS     7
LbaGlu53   ...............................MEVSNKKIT     9
LbaGlu54   ............................MMSGACCRLFLI    12
LbaGlu55   ..........................MKTLSLYDILLLIL    14
LbaGlu56   .................................MGLFSFS     7
LbaGlu57   ...............................MEMFIK..T     7
Consensus
```

```
LbaGlu01   PPSETVKLIQSTS.IEKVRLYGADPAIIKALAN.......    73
LbaGlu05   SPYQSIELIKTMK.AGKVKLYDANPEILRLLSG.......    46
LbaGlu06   PPSQVAQFLKDKTVIDRIKIFDINPDILRAFAN.......    70
LbaGlu08   PPDIVVKLLKENG.FNKVKLFEADPGALKALGR.......    82
LbaGlu12   PPTEVATFIKEQTTINKIKIFDANPDIIRAFAD.......    67
LbaGlu15   EPKIVVQMLKNNG.IKKVKLFDADKSIMNALRGTRSGAIS   199
LbaGlu16   IPTKSVDLIKSLK.AKKVKIYDTNPEILKALKN.......    71
LbaGlu17   ........................................     0
LbaGlu18   VPSMVVDLLLQNG.IPELKLFSPSQYVLPAFAN.......    38
LbaGlu20   VPSVVVDLLLQNK.IPALRLMTSGYDIIEVFSA.......    67
LbaGlu21   PPNSVVKMLKENG.FDKVKLFEADEVILNAIVG.......    74
LbaGlu22   QPSTIVQMLKDNK.IDKVKLFDSDHWIVKYFAG.......    68
LbaGlu23   SPTQVVALLKAQR.IHKVRLFDADQAMLLALAN.......    75
LbaGlu24   VPSMVVDLLLQNK.IPALRLMTSGYDIVEVFAD.......    66
LbaGlu25   HPTQVVALLKAQQ.IRKVRLFNADRGMLLALAN.......    70
LbaGlu26   VPSMVVDLLLQNK.IPALRLFTSASDIIQIFSG.......    69
LbaGlu27   SPSIVVDLLKENK.IQKVKLFDADQIVMKGLMG.......    67
LbaGlu29   PPEIIVKMLKDSG.FQKVKLFDADYDTLKALGN.......    71
LbaGlu30   SASKVIALLKSQG.VEKVKVYDADPAVLKALSG.......    70
LbaGlu31   TIVPLQSFPSTGI...........................    42
LbaGlu36   SPADIVAFLQLQK.ITHIRLYDADPDILKALAK.......    86
LbaGlu37   PAPIVVALLKARQ.ITKVRLFNADAHMLKALAN.......    72
LbaGlu41   PPSKVVELLKANN.INKVKLFDTNSEILEALSG.......    64
LbaGlu46   SPKKVAQLLQSTI.IDKVKIYDTNPEILEAFSN.......    70
LbaGlu47   PPNIVVKMLKDNG.IQKVKLFDADSDIFKALSG.......    71
LbaGlu48   PPKIVVQMLKDNG.IQKVKLFDADQSTMSALAG.......    62
LbaGlu49   SPAQVVQLLKTQG.VNKVKLYDTDSNVLKSLSG.......    69
LbaGlu50   TPDKAVQLIQMHN.IKYVRIYDSNIQVLKAFAN.......    70
LbaGlu51   PPKIVVKMIQENG.IKKVKLFNNDETIINALAG.......    68
LbaGlu52   VPSMVVDLLLQNR.ISGVRIFQPSFNVLDALAD.......    73
LbaGlu53   PPSQVAQFLKDKTLIDKIKIFDLNPDILRAFAN.......    77
LbaGlu54   PPESVVKMLKENG.FEKVKLFEADDKILSALIG.......    72
LbaGlu55   SPTQVVALLKAQQ.IRKVRLFDADRAMLLALAN.......    82
LbaGlu56   PPEIVVRLLKDNG.IQKVKLFDADYETLKALGK.......    73
LbaGlu57   PPSQVAQFIKNKTLIDKVKIFDMNPDIIRAFAN.......    75
Consensus

LbaGlu01   TGIGIMIGASNGDIPALASDPNFAGQWVSSNVLAYY....   109
LbaGlu05   TNLHVSIMVPNDQISAVAASQSAANRWVRDNVLSYY....    82
LbaGlu06   TGISVIVIVPNGEIPNLM.DLAYAKRYVESNIKPFY....   105
LbaGlu08   SGVQVMVGIPNDMLATLSGSVRAAEQWVQKNVSSFIS..K   120
LbaGlu12   SKVWVTITVPNGDILIVS.KPEGAQLWVEENVVPYY....   102
LbaGlu15   TDIEVMVAIPNDLLLTM.TDYDRAKDWVRHNISRYNF..K   236
LbaGlu16   TDLQISVMVPNELINNISTNQTLADQWVKTNVVPFY....   107
LbaGlu17   ........................................     0
LbaGlu18   SSIGVSVAFQENQLRWMN.NSKDIHDWIEKYVKRHV...D    74
LbaGlu20   TNISISITLGNQFVWQAH.RKDLAYVWVNDRIKDPI...N   103
LbaGlu21   SDIEVMLAIPNYMLKDLSSDPLIAASWVDANVTAYAY..T   112
LbaGlu22   TGIEVMIGIPNNQLGKFADDYDFAKEWVKNNVSTHLY..N   106
LbaGlu23   TGIRVIVSVPNDQLLGIGQSNSTAANWVSRNVLSHV....   111
LbaGlu24   TNISISVTLNNQFVWQAN.RKELAYAWINDRIKAPI...N   102
LbaGlu25   TGIKVAVIVPNEQLLGVGQSNSTAANWVAQNVVSHY....   106
LbaGlu26   TNISISICLGNFVVNNLN.RKDLAYTWINDRVNEPI...K   105
LbaGlu27   SGIEVMVGIPNDMLALISSSTNAADLWVAQNVSRYMV..K   105
LbaGlu29   SRIEFMVGIPNDMLTSLA.SLKVAEKWVSKNISVHLS..D   108
LbaGlu30   SGIKVIVDLPNELLYNAAKRQSFAYSWVQKNVAAYY....   106
LbaGlu31   ..ISIAVSLPIEDLYGVSTSVLEAEKWLRTNVLAHY....    76
LbaGlu36   TKIRVIISVPNNQIIAIGSSNTTAAAWIGRNVAAYY....   122
LbaGlu37   ASIEVIIGVTNEEVLRIGESPSAAANWVNQNVAAYM....   108
LbaGlu41   SNIDVTVGIPNTMLRSLNSSLKSAHSWVHNNLTRYF...S   101
LbaGlu46   TGIDIIVAVENSNVTNLSANQSAADEWFSTRILPFI....   106
LbaGlu47   SGIEVMVGIPNDLLWSLANSLGAAEKWVEKNLSSYVS..S   109
LbaGlu48   SDIEVMVAIPNDQLSAM.NDYDRAKTWVRRNVTRYNF..K    99
LbaGlu49   SNISVIVALPNEQLSDAASKQSFTDSWIQSNILSYY....   105
LbaGlu50   TGIELMIGIPNSDLLPFSQFQSNANTWLKNSILPYY....   106
LbaGlu51   TGIEVMVGISNQLLKDIV.NPDIAKKWVKENVTRYEPKSP   107
LbaGlu52   TNIGVIITIQARFLQTMK.EKKQLDDFIYDFVKQYI...D   109
LbaGlu53   TGISVIVIVPNGEISKLL.DIGYAKSYVEANIKPFY....   112
LbaGlu54   SDIEVMLAIPNYMLQDMSTDPGLAASWIDANVSTYAY..T   110
LbaGlu55   TGIRVIVSVPNDQLLGIGQSNATAANWVSRNILSHV....   118
LbaGlu56   SGIEVMVGIPNDMLSTLG.SLKAAEKWVSKNVSVHIS..N   110
LbaGlu57   TGISVIVTIPNGEILKLL.DIGYAKNYVERNIKPFF....   110
Consensus
```

```
LbaGlu01    PASKIIVINVGNEVVISGDQ.N.....LIPQLLPAMQNVQ    143
LbaGlu05    PNTMIRYIIVGNEVLSNKDDQS.....LWYDIVPAMRNIK    117
LbaGlu06    PQTKIIVILIGNEVLHWDTP.E.....VQNKIVPAMKVFY    139
LbaGlu08    NGVDIRYVAVGNEPFLKAYKDM.....FLNTTLPAIENVQ    155
LbaGlu12    PHTRIHRICIGNEVMATGDK.N.....LIAHIVPAMRAIH    136
LbaGlu15    DGVNIKYVAVGNEPFLSAYNNS.....FLNSTFPALQNIQ    271
LbaGlu16    PDTLIRYLIVGNEILSSPPN.T.....TWFNIVPAIRKIR    141
LbaGlu17    ........................................      0
LbaGlu18    HGVDIRYLYVGNEPFSRK.YKQ.....RTFGDVLYFMGEA    108
LbaGlu20    KGVKIVEVIVGSEPFSNTFLKE.....AKSDNMASVLRLM    138
LbaGlu21    NGVKIRYVAVGNEPFLETYNGT.....YLQYILPALKNVQ    147
LbaGlu22    GGVNIKYIAVGNEPFLKSYNGS.....FMKSTFPALQNVQ    141
LbaGlu23    PATNITAIAIGSEVLTTLPN.......AAFVIVSAMKFIH    144
LbaGlu24    KGVKIVELAVGSEPFSNTFYKD.....KRYDHVVGVLRLM    137
LbaGlu25    PATNITTICVGSEVLSALPN.......AAPIIVNALKFVH    139
LbaGlu26    KGVKIVELSIGAEPFSNTFLKQ.....ATNYEVVNVIEIF    140
LbaGlu27    GGVNIKYVAVGNEPFLTSYSGQ.....YQSYVVPAMTNLQ    140
LbaGlu29    NNVNIRYVAVGNEPFLETYNGS.....YLRTTFPALQNVQ    143
LbaGlu30    PSTQIESIAVGNEVFVDPHN.......TTRFIVLAMKNIH    139
LbaGlu31    PATNISTIIVGHTLLCQKDQQN.....KQTLILPSVKNIY    111
LbaGlu36    PQTLITAIAVGDEILTTVPT.......SSPLLMPAIESLY    155
LbaGlu37    PGTNITAIAVGSEVLTAIPN.......AAFVIVPAMNYLH    141
LbaGlu41    NGARIQYIAIGDEPFNQIHGEQ.....FLPFVVGAAENIE    136
LbaGlu46    PATSIVAIAVGNEYLTTDGDGDGDDKLDPNAIVQAMQNLH    146
LbaGlu47    NSVDIKYVAVGNEPFLSQLNGT.....YLPTTFPALQNIQ    144
LbaGlu48    GGVNIKYVAVGNEPFLTSYNNS.....FLNTTFPALQNIQ    134
LbaGlu49    PKTLIEFIAVGNEVFADPNN.......TTRFIVPAMKNVY    138
LbaGlu50    PATKITYITVGAELTEAPNS.......TSTIVVPAMQNVL    139
LbaGlu51    KGVNITIVGVGNEPFLRDYKDT.....LTNVTGPALENIQ    142
LbaGlu52    KGVKFRYLYVGNEPFLKSLYKK.....DRFNNIVHFLNMT    144
LbaGlu53    PQTKIIVIAIGNEILHWETP.E.....VQNKIVPAMRTFY    146
LbaGlu54    RGVKIRYVAVGNEPFLQTYNGT.....YLHCTLPALRNVQ    145
LbaGlu55    PATKITAIAIGSEVLTTLPN.......AAPIIVSAMQFIH    151
LbaGlu56    NPVNIRYVAVGNEPYLTTYNGT.....YLKTTFPAMQNIQ    145
LbaGlu57    PQTKIDIIAVGNEVLLLQGP.E.....IYTKIVPAMKILY    144
Consensus
```

```
LbaGlu01    NAINAASIGGKIPVSIVHAMSILSQSDPP...SSGLFNPV    180
LbaGlu05    NSIDEHNIH.NIKIGTPLAMDILQTSFPP...SSGEFRLD    153
LbaGlu06    QAIGEVGLK.GIPVSSPHSLGILLRSNPP...SAARFRPG    175
LbaGlu08    AALIKAGLARQVPVIVPINAIVYQTDSGV..PSGGDFRTD    193
LbaGlu12    KALLIAGIS.DIQVSTLHSSGILTRSEPP...SSGRFRFV    172
LbaGlu15    NAINQAGVGDSIKATVPLNAIVYFSPESNPVPSAGRFRPD    311
LbaGlu16    CSVKKFGLG.KIPVGTPLAIDMLESSFPP...SNGTFRSE    177
LbaGlu17    ........................................      0
LbaGlu18    RKSLDRHNLTHIKTTTAHFTDILTNVTKP...SKGDFRED    145
LbaGlu20    RETLDEVGLGYVKTTTGHGIIVLPVTKFP...SEADFRDD    175
LbaGlu21    ESINKAGLGAEVKATIPFNADIYYSPESNQVPSAGDFRPE    187
LbaGlu22    KAINSAGLGDKIKATIPQNAIVYESGNSG..PSQGNFRSD    179
LbaGlu23    SAIVAANLDSKIPVSTPHSSSIVLDSFPP...SQAFFNRS    181
LbaGlu24    RQSLDEMGLGHVRTTTGHGMIVLPVTKFP...SEADFRDN    174
LbaGlu25    SAIVASNLDRQIPVSTPLASSIILDSFPP...SQAFFNHT    176
LbaGlu26    RECLDEMDLGHVITTTAHGMIVLPVTPVP...SESDFRDD    177
LbaGlu27    QSIAKANLARNVKIVVPCNADAYESSLPS....QGTFRPE    176
LbaGlu29    TAIVKAGHGNQVPVIVPLNAIVYESASSL..PSSGDFRAD    181
LbaGlu30    QAIVKYNFHDKIPVSSPIALSALQNSYPS...SAGSFRSE    176
LbaGlu31    YSITRWGLHDEIPVSTSFSSNCLDQDSDMA..........    141
LbaGlu36    SAIVAANLHTQIKISTPNAASIILDPFPP...SQAFFNQS    192
LbaGlu37    KAIVASRLNNQVPVSTPQSMIVIAKAFPP...STATFNSS    178
LbaGlu41    AALTKAKLAGPVKIVVPCSFDAFQSESGS..PSKGHFRAD    174
LbaGlu46    SVLLSRGLARKIPVSTPHSMAVLATSFPP...SSSTFATT    183
LbaGlu47    AALIKAGLGNPVPVTIPLNAIVYESSSSQ..PSTGDFRQD    182
LbaGlu48    NAINEAGLGNSIKATVPLNAIVYFSPDSNPVPSAGRFRKD    174
LbaGlu49    SSIVKYNVASKIPVTSPVALSALGNSYPS...SSGSFKPD    175
LbaGlu50    TALKKAGLHKKIPVSSTHSLGVLSRSFPP...SAGAFNSS    176
LbaGlu51    NAINDAGLGDTTKATVPLNAIVYLSPSWNPVPSAGFFRAD    182
LbaGlu52    RDSLDGFNLRDLPVTTPHFTIVLIPVNKP...SEGDFRED    181
LbaGlu53    QALTQSGIN.TIPVSTPHSLGILLSSNPP...SMARFRPG    182
LbaGlu54    EAINHAGLGPKVKAIVPLNADIYYSPYSNPVPSAGDFRPE    185
LbaGlu55    SAIVATNLASQIPVSTPHSSSIILDSFPP...SQAFFNRS    188
LbaGlu56    TAIVKAGLGNQVPVTCPLNAIVYESANSL..PSGGDFRAD    183
LbaGlu57    QALIQSGIN.TIPVSTPNALGILSKCDFP...SMAKFRPE    180
Consensus
```

```
LbaGlu01   FG..DTILKALLQFHKENGSPLMINPYPFFAYQSDPRPETL   218
LbaGlu05   IPRNNLLLPLLRFLNWTKSYFFIIVYPYFSWSQNPSSISL    193
LbaGlu06   WD.VGILAPMLKFLRETKGPFMVNPYPYFGYDPK....QE    210
LbaGlu08   IH..DLMMKIVKFLSDNGGPLTINIYPFLSLDAD.PGFPV    230
LbaGlu12   YD.FVIFAPMLEFHRETKSPFMICSYPFFGFNDK....TL    207
LbaGlu15   IA..ELMTQIVQFMSKNQAPFIVNIYPFLSLYAN.EHFPI    348
LbaGlu16   IS.EPVMVPLLHFLNKTKSFFFIIVYPYFAWAAQPNVINL    216
LbaGlu17   ........................................      0
LbaGlu18   IK..ELMINLLKFLKETKAPIVINIFPIYIVGS..KQMPL    181
LbaGlu20   IK..GPMLDSLDQFNKTGTPFVLYMFPIHFVKEV.LNYTM    212
LbaGlu21   AR..DLTLQIVQYLYSNNAPFVVNIYPFLSLYGN.IYFPL    224
LbaGlu22   IR..DLMQKICRFFKDNNAPFIVNIYPFLSLHEN.KNFPI    216
LbaGlu23   LD..FVMVPLLNFLKDTGSHLMINVYPYYDYMKSDSMIPL    219
LbaGlu24   IK..GPMLDLLYEFNKTGTPFLIYMFPIHFVKIV.LNYTM    211
LbaGlu25   VK..FVLLPLLKFLQSTNSFFMINVYPYYDYMQSNGVIPL    214
LbaGlu26   IK..GLMLQSLNFFNRTGTTFLINLFPIQMIQEV.LNYPI    214
LbaGlu27   LT..QIITQMVSLLNSNGSPFIVNIYPFLSLYGN.SDFPQ    213
LbaGlu29   IH..GFAIQIVKFLSDNGCPFIINIYPFISLYTD.SNFPV    218
LbaGlu30   LI.EFVIKPMLDFLRQTGSYLMVNCYPFFAYESNSIVIPL    215
LbaGlu31   ...EQYIKPLLDFFQCVNAPYIVSLTRDLIVKI.......    171
LbaGlu36   MS..SVISKLLQFLSRTQSPLMMNLYPYYVFMQNKGVVPI    230
LbaGlu37   WN..STIFQILQFLKNTNSYYMLNAYPYYEYVHSAGIFPI    216
LbaGlu41   VN..RTMAELLRFLSKHQSPFFVNISPFFSYRNN.KNISL    211
LbaGlu46   LL..PTMTSIVTLLADTNSAFMVNAYPYFAYRDNPSIVNL    221
LbaGlu47   IR..DLMVNIVKFLNDNGGAFIVNIYPFISLYND.PNFPA    219
LbaGlu48   IN..DLMTQIVQFMSQNNAPFIVNIYPFLSLYAN.EHFPV    211
LbaGlu49   LI.EFVIKPMLSFLKQTGSPLMVNIYPFFAYIANTDTISL    214
LbaGlu50   HA..YFLKPMLEFLAENKSPFMIDLYPYYAYRDSSTNVSL    214
LbaGlu51   IV..DPLNYILPVLDKNNAPFMVNIYPFLSLFYGNGAFPF    220
LbaGlu52   IK..DEMIEFLDYINKTGGPFVINVFPIYTIYR..YGFDA    217
LbaGlu53   WD.VGILQPMLQFLRETKGPFMVNPYPYFGYNPQ....QV    217
LbaGlu54   IR..DLAIQIVQYLHSNDAPFVVNIYPFLSLYEN.NYFPL    222
LbaGlu55   WD..FVVIPMLKFLQSTESYLMINVYPYYVYKQSNDAIPL    226
LbaGlu56   IH..GYVSQIVKFLSDNGCPFTINIYPFISLYID.SDFPV    220
LbaGlu57   WD.VSIIVPMLQFLRETNSPFMVNPYPYFDYDPQ....RS    215
Consensus

LbaGlu01   AFCLFQPNA...GFVDSGSGIKYMNMFDAQVDGVRSALNA    255
LbaGlu05   DFALFKG...THTYIDPDSGYVYTNLLDQMLDSVIFAMQK    230
LbaGlu06   DFLLFRK...NKGVYDRFSKRWYNNSFDMLLDAVYMSMVR    247
LbaGlu08   DFAFFSGT....SAFVVDGSISYTNVFDANYDTIVWALEK    266
LbaGlu12   DYALFKP...NNGVYDPVTGMNYTNMFDAQLDAVYSAMKF    244
LbaGlu15   DFAFFDGG....NTFVNDNGVEYTNVFDANFDTLYSALKA    384
LbaGlu16   DYALLESK..NVIVKDPGSGLIYTNLLDQMIDAVYFAMKR    254
LbaGlu17   ........................................      0
LbaGlu18   DFAFFDERS...NYTIKDGPHIYKNIFTLTYDTIVSALTK    218
LbaGlu20   EFAFLDNKS...GFKIQDGNFTYTNAVELMIDSLAWALKK    249
LbaGlu21   DFAFFDGSK...VKFVRDGGNVYTNVFDANFDTIVWSLKK    261
LbaGlu22   EFAFFDGQ....SKFVRDNGISYTNMFDANLDTIVVSMKK    252
LbaGlu23   DYALFRPLPPNKEAVDRNTHLHYTNVFDAVVDAAYFSMSY    259
LbaGlu24   EFAFLDNKS...GFKIQDDNATYTNAVEFMIDSLAWAIKK    248
LbaGlu25   DYALFKPLAANKEAVDSNTLLHYTNVFDAMIDAAYFAMLD    254
LbaGlu26   EFAFFDNKS...KFKIQDGNFTYTNVVELMIDSIAWAVKK    251
LbaGlu27   DYAFFEGT....THAVTDGPNVYYNAFDGNLDTLIAALAK    249
LbaGlu29   EYAFFDGK....ATPIIDGGAIYTNMFDANHDTIVWALKK    254
LbaGlu30   DYALFHE...NPGVVDAGNGLRYFSLFDAQIDAVNAALSG    252
LbaGlu31   ...........................DTLLNTHLGSMKK    184
LbaGlu36   DNSLFKPLTPSKEMVDPNTLLHYTNVFDAMIDSVYFSMKN    270
LbaGlu37   EYALFQPLSAVKQIVDPNTLFHYESMFDAIVDATYNSMEA    256
LbaGlu41   DFALFKET....ARPHKDNRRTYKNSFDLSYDTLISALSS    247
LbaGlu46   EYALLGNA....TGVRDPKGYVYNNMLDAQIDAIRSAINA    257
LbaGlu47   DYAFFDGY....SSPIDDNGKIYNNVFDANHDTLLWALQK    255
LbaGlu48   DFAFFDGA....SNPIVDGGVTYANVFDANFDTIVSALKA    247
LbaGlu49   DYALFRD...NKGQTDPKNGIVYKSLFEAQIDAVYAAMKA    251
LbaGlu50   DYALFEASS...FVIDPNTGLLYTNMFDAQLDAINYALMA    251
LbaGlu51   DYAFFDGVS...NPLKDKDGVEYTNCFDANLDTCAAALAG    257
LbaGlu52   DFAFFDNKS...KFKIIDGNNTYNNLFTFIYDTIVCALTK    254
LbaGlu53   DFLLFKQ...NPGVFDKFSKKMYTNMFDMLLDAVYMSMKR    254
LbaGlu54   EYAFFNAS....NKPINDGNYLYTNVFDANFDTLAWSLKK    258
LbaGlu55   DYALFRPLPPNKEAVDSNTLLHYTNVFDAIVDAAYFAMSY    266
LbaGlu56   EYAFFDGN....ATPLNDGGTMYTNMFDANHDTIVWALQK    256
LbaGlu57   DFFLFRQ...NKGIFDNVIKKLYTNMFDMILIVVYLSMKK    252
Consensus
```

```
LbaGlu01    WGFKE..IQIVVAETGWPYKGDPNEVGPSVDNAKAYNGNL    293
LbaGlu05    LGFYN..IRLAIAETGWPNGGDYDEIGANVYNAATYNRNL    268
LbaGlu06    LKYPD..VEIVAAETGWPSAGESYEPQCIVENAASYNGGL    285
LbaGlu08    NGFGS..LPIIVGEIGWPTDGDS...NANIEYARKFNQGL    301
LbaGlu12    LGYDD..VDIVVAETGWPSSADPNQPGVSLENAVSYSVNL    282
LbaGlu15    VGYGN..MPIIVGEVGWPTDGDK...NANLNNAYRFYRGL    419
LbaGlu16    VGYPD..VRLFIAETGWPNAGGMDQIGANIFNAATYNRNV    292
LbaGlu17    ........................................      0
LbaGlu18    AGYPD..MQIIIGQIGWPTDGYP...NANPKNAERFHRGL    253
LbaGlu20    AGYPN..IKIVIGQIGWPTDGYP...HANVKNAERFHKGF    284
LbaGlu21    AGFPD..MKIVVGEVGWPTDGDK...NANIENAMRFNQGL    296
LbaGlu22    ANCQG..VKVIVGEIGWPTDGDL...YANVTLAKKFYSGF    287
LbaGlu23    LNFTN..IPIMVTESGWPSKGDSSEPDASLDNANTYNSNL    297
LbaGlu24    AGYPK..MKIMVGQIGWPTDGYP...HATAKNAERFHKGL    283
LbaGlu25    VNFTN..IPVMVTESGWPSLGDSKEPDATVDNANTYNSNL    292
LbaGlu26    AGYPN..MKIMIGQIGWPTDGYP...HAHIKNAERFHKGL    286
LbaGlu27    IGYGQ..MPIVIGEIGWPTDGAV...GANVTAAPVFNQGL    284
LbaGlu29    NGFGN..LPIMVGEIGWPTDGDR...NANVQLAQRFNQGF    289
LbaGlu30    LKYDD..IKMVVTETGWPSKGDETEKGASVDNAASYNGNL    290
LbaGlu31    LGNYNLNNIFLISEN..PKQGRP..LSRKLSTFDSKYTNF    220
LbaGlu36    LNVTD..VAVIVTESGWPSKGDSKEPYATIDNADTYNSNL    308
LbaGlu37    FNFSD..IPIVVTETGWPWDGGSKEPDATKENAETFNNNL    294
LbaGlu41    VGFDK..MDIVIGQIGWPTDGAA...DATSFNAQVFMKGL    282
LbaGlu46    LGFGNRSVQIMVSESGWPSKGDAGETAATTQNARTYNSRL    297
LbaGlu47    NGFPN..MSIIIGEIGWPTDGDN...NANLKAAQRFNQGF    290
LbaGlu48    AGVGN..MTIIVGEVGWPTDGDK...NANVNLAYRFYKGL    282
LbaGlu49    LNFDD..VAMAISETGWPSKGDESEVGATAANAAAYNGNL    289
LbaGlu50    LNFKT..VNIMVTETGWPSKGSPKETAATPDNAQTYNTNL    289
LbaGlu51    AGYSN..MTITVGEMGWPTDGNP...YANVTLAEKFYKGF    292
LbaGlu52    VGYGD..MEIIIGQIGWPTDGYI...GANVENAERFYRGL    289
LbaGlu53    LGYDD..VEIIAAETGWPSLGESFEPQCIVENAASYNGGL    292
LbaGlu54    AGFPD..MKIIVGEVGWPTDGNK...NANTENAKRFNQGL    293
LbaGlu55    LNFTN..IPITVTESGWPSKGDS...DATLDNANTYNSNL    301
LbaGlu56    NGFGN..VPIIIGEIGWPTDGDR...NANAQLAQRFNQGF    291
LbaGlu57    LGYDD..VEIVASETGWSSIGDNFEPQCSVENAASYNRGL    290
Consensus


LbaGlu01    ISHLRSMN..GTPLMPGIS.VDTYIFALYDEDLKPGPGS.    329
LbaGlu05    IRRITSQPSIGTPARPGTA.IPTFIFSLYDENQKGGPGT.    306
LbaGlu06    LRKYNSGT..GTPLMPKRK.IETYIFALFNENTKPGSIA.    321
LbaGlu08    LDRITRGI..GTPKRPTP..PDIYLFGLIDEDAKSILPGN    337
LbaGlu12    VKEVNSGV..GTPLMPNRT.FETYVFSLFNED........    311
LbaGlu15    FTKLASNK..GTPLKPGY..IEVYLFGLIDEDAKSSAPGN    455
LbaGlu16    VKKFTAKPFVGTPTKPGVV.VPTLLFALYNENNKPGPGT.    330
LbaGlu17    .......................MYLHIITDKHNSRLTFGA     18
LbaGlu18    LRYLKRNE..GTPLHPNKT.MIVYITGLSDENKIITEWGE    290
LbaGlu20    LKFLASNK..GTPLRPG.P.IDAFLHSLSDENQFRTMFGA    320
LbaGlu21    IKHCLSGQ..GTPARKGKK.IEVYLFSLIDENAKSIAPGN    333
LbaGlu22    FKKMATKK..GTPLYPGY..IEYYLFSLTDENQKSIAPGS    323
LbaGlu23    IKEVLNNT..GTPKHPGIA.VSTYIYELYNEDLRPGSIS.    333
LbaGlu24    LKFLASNK..GTPLRPG.P.IHMFLHSLTDENLCNTTFGA    319
LbaGlu25    IKEVLNKT..GTPKHPGMA.VSTYIYELYNEDNKAGPLS.    328
LbaGlu26    LKFLASNK..GTPLRPG.P.IDAYLHSLSDENEFKTMFGA    322
LbaGlu27    VKEVLSNK..GTPLRPGVPPMIVYLFSLLDEDAKSVLPGN    322
LbaGlu29    MAHISDGK..GTPMRPGP..IIVYLFSLIDEDAKSIQPGN    325
LbaGlu30    VKRILTGG..GTPLRPKEE.LTVFLFAIFNENKKPGPTS.    326
LbaGlu31    PTLPTPLAPNHSPAFAANSPLPPLIGNISPPPSSLPFAPE    260
LbaGlu36    IKHTIDRS..GTPLHPEIT.SSVYIYELFNEDLRSPIVS.    344
LbaGlu37    IRFVSNDT..GPPSQPKNP.INTFIYEMFNEDKRPGPIS.    330
LbaGlu41    VDHLHSRA..GTPLRPKEPPTETFIYSLFDEDQRNLTSGN    320
LbaGlu46    VERAQSNK..GTPMSPKDG.IDIFVFALFNENKKQGGTG.    333
LbaGlu47    MTHILGGK..GTPMRPGP..IDAYLFSLIDEDAKSIQPGN    326
LbaGlu48    LTRLAANT..GTPLRPGY..IEVYLFGLIDEDGKSTAPGN    318
LbaGlu49    VRFVLTGS..GTPLKPNEP.LNVYLFALFNENQKPGPTS.    325
LbaGlu50    IREVVNNT..GTPAKPGG.AVIVYVFSLFNENRKPGLAS.    325
LbaGlu51    VSYLSKGK..GSPRRPG..YIEAYIFALFDEDRKSTLPGN    328
LbaGlu52    LKYIARKE..GTPLRPNWD.ISMYLQSLTDENKNELEYGP    326
LbaGlu53    LRKYVTGA..GTPLMPHRK.IETYIFDLFNENSKTGSNA.    328
LbaGlu54    IQHALSGE..GTPARKGK..IIVYLFSLIDENTKSIAPGS    329
LbaGlu55    IKEVLNNT..GTPKHPGIA.VSTYIYELYNEDLRQGSTS.    337
LbaGlu56    MQHISAGK..GTPMRPGP..VDAYLFSLIDEDAKSVRPGN    327
LbaGlu57    IRKYMSNV..GTPLMPNRK.IEIYIFSLFNENIKKGNGS.    326
Consensus
```

```
LbaGlu01   .ERSFGLFKPDLSTTYEVGLSKNAQTPTTEVTEVTPAPA.   367
LbaGlu05   .ERHWGLLHPNGRPIYDIDLTGEIPEAEFSKLP.......   338
LbaGlu06   .EKNFGLFRPDFSAVYNIGVLKEDQAHPTPAPAPKNGG..   358
LbaGlu08   FERHWGIFYYDGAIKYKLNLG..N................   359
LbaGlu12   .ERNFGLFRPDFTEVYIVGILRNA................   334
LbaGlu15   FERHWGIFRYDGQPKFPMDISGQG................   479
LbaGlu16   .ERHFGLLYPNGTNVYGIDLSGKTSESEYTPLP.......   362
LbaGlu17   FKRHWGIYETDGNPKYKIDFSLQD................    42
LbaGlu18   YQRHWGIYRHDGSPKYKIDFSMQD................   314
LbaGlu20   FQRHWGIYESDGNPKYKIDFSLQD................   344
LbaGlu21   FERHWGMFEFDGKPKYELDLSGMMQ...............   358
LbaGlu22   FERHWGIFKYDGQPKFAMDLSGQG................   347
LbaGlu23   .EKNWGLFDSSGMEVYTLHLTGSGM...............   357
LbaGlu24   FQRHWGIYQSNGNPKYKIDFSLQD................   343
LbaGlu25   .EKNWGLFNNNGTEVYILRLTESGS...............   352
LbaGlu26   FQRHWGIYQADGNPKYKIDFSLQD................   346
LbaGlu27   FERHWGIFSFDGQSKYPLNLGNGL................   346
LbaGlu29   FERHWGIFAYDGQPKYSLNLGTTN................   349
LbaGlu30   .ERNFGLFYPNEKPVYDIPLTMEGLKHYVDRPP.......   358
LbaGlu31   MPEVFNPISPFNGPHLPPCIPSHGGGG.............   287
LbaGlu36   .EAHWGLFHGNSTEVYLLHVSGSGT...............   368
LbaGlu37   .EKSWGIFSTNGTEVYSISLGSSG................   353
LbaGlu41   FERHWGLFTFDGQAKYQLDLGQGL................   344
LbaGlu46   .ERNFGIFNADGSKVYIVDLSCQFCSGNS...........   361
LbaGlu47   FERHWGIFYFDGTPKYSLSLGGNN................   350
LbaGlu48   FERHWGIFRYDGQPKFAMDLTGQG................   342
LbaGlu49   .ERNYGLFYPNQQFVYNIPLTKEGLESGAPTVNNGSKST.   363
LbaGlu50   .ERNWGLFFPDQTSVYNLDFTGKG................   348
LbaGlu51   FETHWGLYYDDGTPKFPLDLNGKN................   352
LbaGlu52   YQRHWGIYKLDGQPKYKIDFTMQD................   350
LbaGlu53   .ERNFGLFRPDFTEVYNIGIMKGQPLLPMEVQPKPLPQPA   367
LbaGlu54   FERHWGIFEFDGKPKYELDLSGLK................   353
LbaGlu55   .EKNWGLFYPSGAEVYILHLTGSGT...............   361
LbaGlu56   FERHWGILTYDGLPKYILNLGTTN................   351
LbaGlu57   .ERNFGLFRPDFTEVYNIGIMKGE...PLEVLP.......   355
Consensus

LbaGlu01   ....................TTPTTPTTPATEVTPAPKPT   387
LbaGlu05   ...............................GPKNNGPFH   347
LbaGlu06   ....................GNKDKPKSKSEVAAPPAQ..   376
LbaGlu08   ...........................KRSVIAAKGVRYL   372
LbaGlu12   ...........................QASPPAMAPEI..   345
LbaGlu15   ..........................QERHLIAAKNVQYL   493
LbaGlu16   ...............................RPRNNEPYK   371
LbaGlu17   ..........................RDKYPTEAKGIVKM    56
LbaGlu18   ..........................HNSEPTTAKGIVKM   328
LbaGlu20   ..........................RDEYPTQAKGIVKM   358
LbaGlu21   ..........................KDKGIVAVEGVNYM   372
LbaGlu22   ..........................HETMEVGAKNIKYL   361
LbaGlu23   .................................VLANYNT   364
LbaGlu24   ..........................RDDYPTQAKGIVKM   357
LbaGlu25   .................................MEANDTS   359
LbaGlu26   ..........................RDEYPTQAKGIVTM   360
LbaGlu27   ..............................LKNAKIVQYL   356
LbaGlu29   ...........................SGSLIPASNVKYL   362
LbaGlu30   ....................VAGNHRMQKGKKGNVTA.LA   377
LbaGlu31   ..........................GEVGAPAPGGVHGN   301
LbaGlu36   .................................FLANDTT   375
LbaGlu37   ..................................GISDNS   359
LbaGlu41   ............................RNLRNAQNVHYL   356
LbaGlu46   ..........................EKMGGFGEKLSLRG   375
LbaGlu47   ...........................RGGIVPASGVRYL   363
LbaGlu48   ..........................QDKFIVGAQNVEYL   356
LbaGlu49   ..................VVTAPTSSPLPAPIGIVEASKA   385
LbaGlu50   ............................VVDMTGNTTGSN   360
LbaGlu51   ............................KTLASVPNVEKL   364
LbaGlu52   ..........................RDIKPSVAKGIVLL   364
LbaGlu53   LPQPQQPRAPRNRAKPGPILPTQPGPKLPAQPLPKPAAPM   407
LbaGlu54   ..........................KHKSLAAVEGVSYM   367
LbaGlu55   .................................VLANDTT   368
LbaGlu56   ...........................SGSIVPARNVKYL   364
LbaGlu57   ....................PQKPGPRLPAQPLPKPAAFL   375
Consensus
```

```
LbaGlu01    VASWCMPKPG.TPDSELQANLDYACGMSGINCNPIQAGGP    426
LbaGlu05    GKLWCVVTKDFVNEFDLGQALEFACRR.NGTCDEIAPGRS    386
LbaGlu06    NKKFCMPFVE.ATDAQLQSNINYVCS.QGVDCTPIQVGGP    414
LbaGlu08    ARKWCVMAPDANVKD.PNLADSINYACSYADCTSLGYGSS    411
LbaGlu12    GKKWCVPKAD.ASDAALQSNIDFVCS.SGIDCQPIQDGGP    383
LbaGlu15    PKRWCMFNPNAKYYNMSKLADNIDYACSHSDCTALGYGSS    533
LbaGlu16    GKLWCVVGRK.ANASEIVGALTYACGQGNRTCDEIQPGGK    410
LbaGlu17    PSRWCKFNGDKSN..MNSVNKNYNLACEKADCTVLEVGAS     94
LbaGlu18    PNRWCMYNENIFNGSKEFVMEFVNYACSKSDCTLLSPGAT    368
LbaGlu20    PNRWCKFNGDRSN..MNIVNKNYDLACNEADCTRLEKGAS    396
LbaGlu21    QKKWCVLKPLNVVDNAEDLPKNVDYACSLSDCTALGYGSS    412
LbaGlu22    ENKWCVLNKYAE..DIGKLPSSVQYACSRSDCTAMSYGGS    399
LbaGlu23    NQTYCVAKEN.AEKKMVQAALDWACGPGFVDCTPLLQENP    403
LbaGlu24    PSRWCKFNGDKSN..MNIVNKNYYLACKEADCTVLEKGAS    395
LbaGlu25    NNTYCAAKEG.VDSKMLQAALDWACGPGFVDCAPLLQGEP    398
LbaGlu26    PNRWCTFVGDKSD..MDIVKKNYNFACEAADCTTMEEGPS    398
LbaGlu27    PYRWCVANPT...KNLAFVTNFVKLACTYADCTTLNYGGS    393
LbaGlu29    ESKWCVLKPSAKLDD.PQVAPSVSYACAHADCTSLGYETS    401
LbaGlu30    GQTWCVASGE.SGKDNLQAALDYACGEGGADCRSIQPGST    416
LbaGlu31    EGLWCVAKPS.VPPETLQEALDYACGEGDADCEDISPTGS    340
LbaGlu36    NQTYCIAMDG.IDKKTLQAALDWACGPGRANCSEIQPGES    414
LbaGlu37    SAVFCVAKSG.ADENKLQDGINWACGQGRANCSAIQSGKP    398
LbaGlu41    SSKWCVVNNN...QNLSNATARALEACSSADCSVLSPGGS    393
LbaGlu46    PSVWCVAKPH.ADEFVVQAVLDFCCGPGGVDCREIYENGN    414
LbaGlu47    DRKWCVLSPSASLDD.PQLADSVGYACSHADCTSLGHGTS    402
LbaGlu48    SKKWCMLNPNAK..DLSKLADNINYACTFSDCTALGYGSS    394
LbaGlu49    VNTWCVANEK.ATREKLQAALDYACGQGGADCHPIQPGAT    424
LbaGlu50    GTSWCIASAS.ASEADLKNALSWACGSGNVDCSAIQPSQP    399
LbaGlu51    PKKWCVIKPD.....VKNFTALMSYACDRADCTPLTNGSS    399
LbaGlu52    PSRWCVFDGKTDH.NETIVLQDYEYACYTSDCSSLGAGAT    403
LbaGlu53    GKKFCMPKPQ.ATDAQLQASLDWACTNQGVDCGFVQAGGP    446
LbaGlu54    HKRWCMLKPRHALN...DLGKSIDYACSLSDCTALGYGSS    404
LbaGlu55    NQTYCVAREN.ADKKMVQAALDWACGPGFVDCNPLLQGNP    407
LbaGlu56    ERKWCVLKPNAFVDD.PQIAPSMSYACGLADCTSLGYQTS    403
LbaGlu57    GKKFCMPKAN.ATDAQLQANLNWACNNQGVDCGFVQAGGP    414
Consensus       c                          c

LbaGlu01    CFEPNTVASHAAYAMNLLYQIAGRNPWNC.DFSQTASLTS    465
LbaGlu05    CYQFVSIVSHANYAFSSYWAKFRKDGETC.HFSGLAVQTT    425
LbaGlu06    CFSPNTIRSHAAFVMNSYFQKEGRNNFNC.DFAGTGVVAN    453
LbaGlu08    CGGLD.AKSNASYAFNMYYQTMNQQKGSCERFHNLSVITT    450
LbaGlu12    CYEPNTIRAHAAYAMNAYYQANGRKMFDC.NFINTGVVTN    422
LbaGlu15    CNNLD.ANGNASYAFNMYYQVQNQLNVSC.DFQGLAMVTD    571
LbaGlu16    CYKPDSIVLHANYAFSSYWAQFKSLGGTC.SFSGLTIPTK    449
LbaGlu17    CGGMS.FESFVSYAFNAYFQMFKQDIDHC.EFDGLGEIVA    132
LbaGlu18    CDNLN.FTWNASFAFNMYFQMNGQKINYC.DFNGYGYITT    406
LbaGlu20    CDGLS.FESKISYAFNAYFQKYKQFVETC.DFDGLGEIVA    434
LbaGlu21    CNHLS.TDGNASYAFNMYYQLKNQNSWDC.DFDELAVVTD    450
LbaGlu22    CNKLD.ADGNVSYAFNMYYQMNGQIVESC.VFDGLAQIVE    437
LbaGlu23    CYEPNTVIAHASYAFDAYYHKMGMADGTC.DFNGVAFVTT    442
LbaGlu24    CGGIS.SESFVSYAFNAYFQKYKQDIDHC.DFEGLGEIVS    433
LbaGlu25    CYEPDNVAAHATFAFDAYYHMMGRAPGTC.DFNGVATITT    437
LbaGlu26    CGKLT.DESKISYAFNAYFQRKQQNEKAC.DFNGLGEITS    436
LbaGlu27    CNGIG.AKGNISYAFNSYYQLQKQNPRSC.DFDGLGMVTF    431
LbaGlu29    CGGLD.ERGNISYAFNSYYQINNQLIVAC.KFSGLGTITK    439
LbaGlu30    CYNPNTLEAHASFAFNSYYQKNGRAMGSC.YFGGAAFIVH    455
LbaGlu31    CYNPDTIVAHASYAFNSYWQKTKSNGGTC.EFGGTAMLIN    379
LbaGlu36    CYLPNIVKNHASYAFDSYYQKEGSSPPSC.DFKGVAMITT    453
LbaGlu37    CYFPDTLQNHASYAYNDYYQRMRSAGGTC.DFDGTATTTT    437
LbaGlu41    CFNLS.WPGNISYAFNSYYQQHDQRADSC.DFGGLGLITT    431
LbaGlu46    CFPPDKIHAHASYAMNAYYQMHGRNYWNC.DFKGTGIVTF    453
LbaGlu47    CADLD.ARGNISYAFNSYYQENDQLPTAC.KFPNLSMVTN    440
LbaGlu48    CNGLD.ANGNASYAFNMYYQVQNQGDFSC.GFQGLGMLTD    432
LbaGlu49    CYNPDSLEAHASYAFNSYYQKKARGTGTC.DFSGAAFVVT    463
LbaGlu50    CFEPDNLASHASYAFNSYYQQNGATDIAC.SFGGVGVRTN    438
LbaGlu51    CQDLS.DAAKASYAVNAYFQSQQQKDESC.DFEGKATITT    437
LbaGlu52    CDRLS.FTQFVSYAYNIRYQTANQNIHKC.ELIGAEISTI    441
LbaGlu53    CFNPNTVRSHAAFVMNSYYQIKGRNIVNC.DFSGSAVIVF    485
LbaGlu54    CNHLS.AQGNASYAFNMYYQFKSQNSLDC.DFQGLAMVTH    442
LbaGlu55    CYEPDSVFAHASYAFDAYYHKMGMADGTC.NFNGVATVTT    446
LbaGlu56    CGGLD.ARGNISYAFNSYYQINNQLDDAC.KFQGLGTVTK    441
LbaGlu57    CFSPNSVRSHAGFVMNSYYQIKGRNDFNC.DFLGSGVIIF    453
Consensus   c                            c
```

```
LbaGlu01   TNPS........YGGCTYPGGNL.................   480
LbaGlu05   IDPS........HGSCKFPYVSL.................   440
LbaGlu06   ADPS........YGTCKYDS....................   465
LbaGlu08   IDPTPPPSKSGNSSHCRFFVMIIVGKHESFVNPGTSSATK   490
LbaGlu12   YDPS........YEECTYAA....................   434
LbaGlu15   KDLS........QGTCNFIIQTGIY..........SFSHK   593
LbaGlu16   RDPS........YGSCKFPSVTL.................   464
LbaGlu17   TNPS........PDSG.....................GPR   143
LbaGlu18   NDPS........TATCRFPIEIL..............SVE   424
LbaGlu20   TNPT........VKDCEFPIEILA............FQDQ   454
LbaGlu21   EDPS........DDKCRFFVMIAVAV...GHSIVVLLHRK   479
LbaGlu22   KNAS........IDSCLFPIGLESA..........GVRIG   459
LbaGlu23   SDPS........NGSCIYPGSGG...RNGTFKNSSPLAPS   471
LbaGlu24   TNPS........TKNCEFFVEILA............FQD.   452
LbaGlu25   TNPS........HGSCVFSSLG....RNGTFLNGTTPAMD   465
LbaGlu26   TNPS........VEECEFFVEILS............FQDQ   456
LbaGlu27   LNPS........IGECRFIVGVSDHAAIDHHAASSGFRLQ   463
LbaGlu29   SDPS........TPSCRFGIMIEPYY......GGAGRRNG   465
LbaGlu30   QQPK........YGSCELPTED..................   469
LbaGlu31   SDPS........YLHCRFILA...................   392
LbaGlu36   TDPS........HGSCTFPGSKPVNNKTSQVENATQASGA   485
LbaGlu37   KDPS........SKTCKFTGSSH...PGGVFPPAAFGPIG   466
LbaGlu41   VNPS........VGTCRFIVQLGT........SNSALHQK   455
LbaGlu46   SDPS........YGRCFYSQQ...................   466
LbaGlu47   TDPSPP......GGTCKFKIMIQASK......PSSDKSNA   468
LbaGlu48   QNIS........QANCNFTIQIAA...........SFSPK   453
LbaGlu49   QHPN........YGSCKFPTGY..................   477
LbaGlu50   KNPS........YDNCLYATTGGKK...AIAGNATAASPS   467
LbaGlu51   NDPS........QGTCNFTIGFKTLTALSTSPSPLPSSDH   469
LbaGlu52   N.PS........TPECEFSIEIL..............TAE   458
LbaGlu53   ADPS........YGTCKYLA....................   497
LbaGlu54   KDPS........DDKCHFFVMID......DRPFVMLLHKN   468
LbaGlu55   SDPS........HGSCIFPGSGG...KNGTVTNGTTLAPS   475
LbaGlu56   SDPS........TGTCRFGLMIQPYY......GGAERKHG   467
LbaGlu57   ADPS........YGTCKYLS....................   465
Consensus

LbaGlu01   ........................................   480
LbaGlu05   ........................................   440
LbaGlu06   ........................................   465
LbaGlu08   IKHFSLALIVIVFILDYTMSLS..................   512
LbaGlu12   ........................................   434
LbaGlu15   VLPGIVVLLSGFAFLLL.......................   610
LbaGlu16   ........................................   464
LbaGlu17   ILRSWVLTSL..............................   153
LbaGlu18   LIDNGVIHQIVASSGEISKVVSNPTILPIGLALSAIVCMI   464
LbaGlu20   VELNGMVLRI..............................   464
LbaGlu21   LLYIIIVAIIVLMIV.........................   494
LbaGlu22   LDAILNIIVGFFLFLALL......................   477
LbaGlu23   SNTTASGCHSQYSND..HIFFSSFIVGLIIFWIVVSL...   506
LbaGlu24   VLSNGMVLRI..............................   462
LbaGlu25   S.TSSSAYPAQYLNN..NSFSTTLMILGILGLSVILL...   499
LbaGlu26   VANNGMVKLRI.............................   467
LbaGlu27   NSWISVLLIIIILWDAWLFLM...................   483
LbaGlu29   CTSAALALILLLCTIL........................   481
LbaGlu30   ........................................   469
LbaGlu31   ........................................   392
LbaGlu36   NTIRYIGAQAGTVDRNLHVLFSVALCLFYYSLIQVKLT..   523
LbaGlu37   AISQSSTIRAPVIMSIVAVFFALLMQM.............   493
LbaGlu41   FTMITIATILLWIVGGGL......................   473
LbaGlu46   ........................................   466
LbaGlu47   FTSKPVNVMFIVVIVSLLSVL...................   489
LbaGlu48   LLPGIITFLTAFTFVLI.......................   470
LbaGlu49   ........................................   477
LbaGlu50   NSSSSSPQRSYQWTQHLIVAFPLFLIVPF...........   496
LbaGlu51   SSSSDAKAPSSSSASPSSKNSLAFASLGVLFIVSLFFM..   507
LbaGlu52   VVDGGSLINKKY............................   470
LbaGlu53   ........................................   497
LbaGlu54   IVYIILAILQGFIVVLLIVS....................   488
LbaGlu55   SNSTSSGCHLQYTREN.QAFLSSLIVGLILHSAAFW....   510
LbaGlu56   YTRIALALILFLWTIL........................   483
LbaGlu57   ........................................   465
Consensus
```

```
LbaGlu01   ........................................   480
LbaGlu05   ........................................   440
LbaGlu06   ........................................   465
LbaGlu08   IKHFSLALIVIVFILDYTMSLS..................   512
LbaGlu12   ........................................   434
LbaGlu15   VLPGIVVLLSGFAFLLL.......................   610
LbaGlu16   ........................................   464
LbaGlu17   ILRSWVLTSL..............................   153
LbaGlu18   LIDNGVIHQIVASSGEISPVVSNPTILPIGLALSAIVCMI   464
LbaGlu20   VELNGMVLRI..............................   464
LbaGlu21   LLYIIIVAIIVLMIV.........................   494
LbaGlu22   LDAILNIIVGFFLFLALL......................   477
LbaGlu23   SNTTASGCHSQYSND..HIFFSSFIVGLIIFWIVVSL...   506
LbaGlu24   VLSNGMVLRI..............................   462
LbaGlu25   S.TSSSAYPAQYLNN..NSFSTTLMILGILGLSVILL...   499
LbaGlu26   VANNGMVKLRI.............................   467
LbaGlu27   NSWISVLLIIIILWDAWLFLM...................   483
LbaGlu29   CTSAALALILLLCTIL........................   481
LbaGlu30   ........................................   469
LbaGlu31   ........................................   392
LbaGlu36   NTIRYIGAQAGIVDRNLFVLFSVALCLFYYSLIQVKLT..   523
LbaGlu37   AISQSSTIRAFVIMSTVAVFFALLMQM.............   493
LbaGlu41   FTMITIATILLWIVGGGL......................   473
LbaGlu46   ........................................   466
LbaGlu47   FTSKFVNVMFIVVIVSLLSVL...................   489
LbaGlu48   LLPGIITFLTAFTFVLL.......................   470
LbaGlu49   ........................................   477
LbaGlu50   NSSSSSPQRSYQWTQHLIVAFPLFLIVPF...........   496
LbaGlu51   SSSSDAKAPSSSSASPSSKNSLAFASLGVLFIVSLFFM..   507
LbaGlu52   VVDGGSLINKKY............................   470
LbaGlu53   ........................................   497
LbaGlu54   IVYIILAILQGFIVVLLIVS....................   488
LbaGlu55   SNSTSSGCHLQYTREN.QAFLSSLIVGLILHSAAFW....   510
LbaGlu56   YTRTALALILFLWTIL........................   483
LbaGlu57   ........................................   465
Consensus
```

Fig S2 X8 domain in *Glu* genes
